# Swedish and Norwegian Police Interviewers' Goals, Tactics, and Emotions When Interviewing Suspects of Child Sexual Abuse

**DOI:** 10.3389/fpsyg.2021.606774

**Published:** 2021-07-09

**Authors:** Mikaela Magnusson, Malin Joleby, Timothy J. Luke, Karl Ask, Marthe Lefsaker Sakrisvold

**Affiliations:** ^1^Department of Psychology, University of Gothenburg, Gothenburg, Sweden; ^2^Research Department, Norwegian Police University College, Oslo, Norway

**Keywords:** police, interrogation, suspect, child sexual abuse, Norway, Sweden, investigative interview, emotion

## Abstract

As the suspect interview is one of the key elements of a police investigation, it has received a great deal of merited attention from the scientific community. However, suspect interviews in child sexual abuse (CSA) investigations is an understudied research area. In the present mixed-methods study, we examine Swedish (*n* = 126) and Norwegian (*n* = 52) police interviewers' self-reported goals, tactics, and emotional experiences when conducting interviews with suspected CSA offenders. The quantitative analyses found associations between the interviewers' self-reported goals, tactics, and emotions during these types of suspect interviews. Interviewers who reported experiencing more negative emotions were more likely to employ confrontational tactics. Specifically, anger was positively associated with the goal of obtaining a confession and with aggressive tactics like raising one's voice and emphasizing the seriousness of the crime. Frustration and disgust displayed similar patterns. Somewhat contrasting these quantitative results, the thematic analysis identified a strong consensus that emotions should not and do not affect the police interviewers' work. Furthermore, the police interviewers described a range of strategies for managing emotions during the interview and for processing their emotional reactions afterwards. The present findings highlight the relevance of emotional processes in CSA suspect interviews and provide an initial exploration of the potentially complex relationship between the goals, tactics, and emotional experiences of police interviewers who question CSA suspects.

## Introduction

Criminal investigations concerning alleged child sexual abuse (CSA) presents a challenge for legal practitioners worldwide. As corroborative evidence is often scarce, the statements provided by child witnesses and suspects typically constitute the primary sources of information (Ernberg et al., 2018). During the last four decades, a considerable amount of research has increased our knowledge of forensic child interviewing during CSA investigations (see e.g., Brubacher et al., 2019). Likewise, a large body of psychological research on suspect interviewing has developed over the last several decades. Much of this literature is focused on risk factors for false confessions (Kassin et al., 2010). Other parts of the literature have been aimed at developing interviewing methods that increase the amount of accurate information obtained while questioning suspects (Mac Giolla and Granhag, 2017). This latter part of the literature has demonstrated that non-accusatory techniques that are focused on gathering information, rather than obtaining confessions, are more effective than confrontational, accusatory techniques (e.g., minimization; Meissner et al., 2014; Luke and Alceste, 2020). However, research is scarce regarding practitioners' experiences of interviewing suspected CSA offenders. In the present study, we aimed to address this gap by examining Swedish and Norwegian police interviewers' self-reported goals, tactics, and emotions when questioning suspects of sexual abuse against a child. Given the low prosecution rate for CSA offenses, there is an urgency for research exploring the current procedures used when investigating these crimes, and their accordance with evidence-based methods (Ernberg et al., 2020).

The lack of research on how to interview suspected CSA offenders poses numerous problems. First, investigating cases of CSA can be challenging, as it includes being exposed to potentially traumatic material or information (Burns et al., 2008; Powell et al., 2014). This may cause these investigations to be emotionally demanding and can even lead to secondary traumatic stress and burnout among professionals (Perez et al., 2010; Bourke and Craun, 2014; Turgoose et al., 2017; Seigfried-Spellar, 2018). Second, perpetrators of CSA differ from non-sex offenders in numerous ways (e.g., more likely to suffer from mental illness, anxiety, low self-esteem, and have greater social deficits, but less likely to have lifestyle instability and antisocial disorder; Whitaker et al., 2008), which may impact on the suitability of different interview tactics. Third, convicted CSA perpetrators have reported a strong internal need to confess to their crimes, compared to general violent offenders or those convicted of raping adults (Gudjonsson and Sigurdsson, 2000). Yet, in Swedish court cases involving CSA, only 31% of defendants admitted guilt during their trial (Magnusson et al., 2018). According to the Pathways model (Ward and Siegert, 2002), there are four psychological mechanisms involved in sexual offending against children. One of the mechanisms—cognitive distortions—may constitute an obstacle for offenders to recognize that they have committed a crime. The interview techniques used by police need to challenge any distorted excuses, encourage offenders to admit to their wrongdoings, while at the same time making sure to minimize the risk of obtaining false confessions. Establishing a respectful and supportive environment is critical to increase the suspect's confidence to engage in the process and provide an honest and detailed response (Read et al., 2009).

The use of different interviewing tactics might vary depending on the type of crime. A study examining 59 police interviews from the UK (Oxburgh et al., 2014) found that suspects of CSA were asked significantly more inappropriate questions compared to suspects of child or adult murder. Furthermore, a Swedish study (Holmberg and Christianson, 2002) that compared self-reported interview experiences between convicted sex offenders (including offenses against both children and adults) and murderers found several differences between crime types. Although only a minority of murderers and sex offenders described their interviewers as aggressive or explicitly confrontational, sex offenders described experiencing less open-minded attitudes, cooperation, and more coercion than did murderers. A majority of the sex offenders experienced stress during the interview, and significantly more sex offenders described a lack of being respected compared to the murderers. Moreover, police interviews marked with dominance (compared to interviews marked with humanity) were associated with a higher proportion of denials. However, it is unclear whether a dominant approach led to denials, or if denying resulted in a negative response by the interviewer. On a similar note, a study based on interviews with convicted sex offenders in Australia reported that sex offenders perceived ethical interviewing and displays of humanity to increase the likelihood of a confession (Kebbell et al., 2010). Despite the findings described above, the use of empathy in police interviews with suspects of sexual abuse has shown no direct effect on the amount of information elicited (Oxburgh and Ost, 2011).

Although we lack data to draw firm conclusions, it is possible that the type of crime influences the way the interviewer conducts the suspect interview, which in turn might affect the likelihood of eliciting information. This raises the question of how police interviewers are affected by the nature of the crime, and how their emotions potentially affect their goals and tactics. Many emotional demands are put on law enforcement and extensive research has examined the causes and consequences of stress and traumatic work experiences (e.g., Toch, 2002; Perez et al., 2010; Seigfried-Spellar, 2018). The focus has mainly been on how work-related stressors impact the psychological health of police officers. Thus, we have identified some important aspects that have received limited attention, namely if and how emotions influence how police interviewers perform their work. This is of particular interest in cases of CSA, as these investigations have been shown to be perceived as especially distressing (Huey and Kalyal, 2017).

Emotions can influence human behavior and decision making in a number of ways that may impact the conduct of suspect interviews. First, specific emotions are associated with cognitive appraisals (Scherer and Moors, 2019) that may shape interviewers' perception of guilt and responsibility. For instance, anger can increase the perception of criminal intent behind ambiguous actions and promote punitiveness (Ask and Pina, 2011) and may increase the attribution of guilt to a suspect under investigation (Sambrano et al., 2020). Second, emotional states influence the type of information processing that an individual is likely to engage in. For instance, it has been shown that police investigators who experience anger tend to process case materials more superficially, and thus rely more on heuristics, than colleagues who experience sadness (Ask and Granhag, 2007). Third, affective states have been found to influence people's strategies in social interactions. For instance, people in a happy (vs. sad) state tend to rely on more direct, less elaborate strategies when spontaneously interacting with, making requests from, negotiating with, and trying to persuade others (Forgas, 2002). Relatedly, specific emotions are associated with distinct action tendencies, which facilitate behavior congruent with the current emotional state (Frijda et al., 1989). For instance, anger is associated with the tendency to approach and confront the source of the emotion (Carver and Harmon-Jones, 2009), whereas fear is associated with avoidance and withdrawal (Lerner and Keltner, 2001).

Studies that directly examine the relationship between emotional experiences and interviewer behavior are scarce. One notable example, highly relevant to the present study, is the recent study by Sambrano et al. (2020). They experimentally induced specific emotions (happiness, sadness, and anger) and observed their influence on mock detectives' preference for interrogation tactics in a hypothetical crime scenario. Across two experiments, while all groups showed a preference for benevolent (empathetic) over hostile (guilt-confirming) tactics, this preference was more pronounced among sad participants than among angry and happy participants. This finding is consistent with the theoretical assumption that emotions associated with certainty appraisals (e.g., anger, happiness) promote confirmation-seeking behaviors, whereas uncertainty-related emotions (e.g., sadness) promote more elaborate, open-minded thinking, and action (Tiedens and Linton, 2001; Lerner and Tiedens, 2006). In sum, these results raise the possibility that the behavior of police interviewers questioning CSA suspects may be influenced by the emotions they experience in response to the nature and content of the case.

The current data collection took place in Sweden and Norway. Although the neighboring countries have much in common with regard to their societal structures, legislations, and criminal investigation practices, there are some key differences in terms of their focus on research-based interviewing (Fahsing et al., 2016). In Norway, a shift toward more research-based police practices began after a number of judicial scandals involving coercive interrogation techniques. In 2002, the Police University College commissioned a national model for investigative interviewing (KREATIV; Fahsing and Rachlew, 2009) that was heavily influenced by the PEACE model from England and Wales (Milne and Bull, 1999). The KREATIV training program focuses on teaching research-based information-gathering techniques for interviewing victims, witnesses, and suspects (Fahsing et al., 2016). The program is now an integral part of the basic education to all Norwegian police officers. In addition, the Norwegian Police University College offers specialized courses in investigative interviewing of adults and the handling of sexual crimes investigations which include some interview training (Detective superintendent C. Tombre, personal communication, August 30, 2019). However, there are no specific guidelines for suspect interviews in CSA cases. While substantial organizational changes have occurred through the implementation of KREATIV, studies are lacking regarding the quality of Norwegian interview practices following the reform.

Unlike Norway, Sweden has not implemented a national interview model that all police interviewers are required to follow. However, Swedish police interviewers are by law not allowed to use threats, coercive techniques including sleep or food deprivation, promises of leniency, or to deceive the suspect (the Swedish Code of Judicial Procedure, chap 23, §6). Furthermore, the police academies across Sweden focus primarily on information-gathering techniques and advise against using accusatory methods (Granhag et al., 2013). Field and laboratory research from suspect interviews involving a variety of different crimes point toward the use of both information-gathering and more confrontational methods among Swedish police interviewers (Granhag and Magnusson, 2017; Mac Giolla and Granhag, 2017). Similar to Norway, Sweden has not implemented any specialized interviewing guidelines for questioning suspected CSA offenders. However, police interviewers can take a course on investigating crimes against children which include some training in interviewing suspects (E. Norrman, course leader, personal communication, September 9, 2020).

The present mixed-methods survey aimed to descriptively examine Swedish and Norwegian police interviewers' self-reported interview goals, tactics, and emotions when questioning suspects in CSA cases. Using a series of quantitative analyses, we aimed at examining the interrelationships between interview goals, tactics, and experienced emotions. Due to differences between countries with regard to the implementation of a research-based investigative interview model, we were also interested in comparing the reports of goals, tactics, and emotions given by Swedish and Norwegian police interviewers. Lastly, we aimed to qualitatively explore how practitioners report managing different types of emotions that may arise during interviews with CSA suspects.

## Materials and Methods

The study was pre-registered on the Open Science Framework (https://osf.io/jtac5/?view_only=e3b14c4113144dd3a857448c4ec6b913). The registration included a recruitment strategy and plan for exploratory analyses. We followed the recruitment plan as specified, but we have deviated from the exploratory analysis plan in order to simplify the presentation of results and to address reviewer comments.

### Participants and Recruitment

A total of 127 Swedish police interviewers responded to the survey. However, one participant did not meet the current inclusion criterion of having experience conducting suspect interviews during the last 5 years. The final Swedish sample thus consisted of 126 participants (105 women, 21 men, *M*_age_ = 45.2 years, *SD* = 9.1), with 101 police officers (2 years of training at the police academy) and 24 civilian officers (academic degree in other relevant fields, for example, criminology or psychology). The interviewers had between 0 and 25 years (*M* = 7.0, *SD* = 5.6) of experience conducting interviews with CSA suspects. The majority (80.2%) had finished the course on investigating crimes against children which includes training on interviewing suspects. The Swedish survey was distributed during a national conference for specialized child interviewers, with a response rate of 79% of the attendees. According to the Prosecution Development Centre Gothenburg (2016), there are around 305 active child interviewers in Sweden. Based on this estimate, our sample would represent 41.3% of the current population of child interviewers in Sweden.

With regard to the Norwegian data collection, a total of 52 police interviewers participated in the survey (45 women, 7 men, *M*_age_ = 40.0 years, *SD* = 8.02). All except three participants, who chose not to report their employment status, had a background as police officers (this requires a bachelor's degree from the Police University College, or equivalent training before the current training program was implemented). The Norwegian participants had between 0 and 24 years (*M* = 8.9, *SD* = 6.5) of experience interviewing suspects in CSA cases. All participants except one (98.1%) had completed the KREATIV training program, and 40.3% had completed the training course on investigating sexual crimes which includes some suspect interview training. Due to varied recruitment methods, we were unable to calculate a response rate for the Norwegian sample.

### Survey

The survey consisted of six different sections. First, the survey included a cover page with information about the research project and the participants' ethical rights (e.g., participation is voluntary and can be withdrawn at any time). All participants were asked to provide their informed consent to take part in the survey. The cover page also included a screening question asking if they had experience conducting interviews with CSA suspects during the last 5 years. Participants who did not have this experience were informed that they were not eligible for participation and thanked for their time. The second section consisted of demographic questions about the participants' experience and previous training in forensic interviewing.

The third section comprised a list of different interview tactics for questioning CSA suspects adapted from the current research literature (see Table 1). The participants were asked to indicate how often they used each tactic. The Likert scales ranged from (1) *Never* to (5) *Always*. The order in which the tactics were presented was reverse counterbalanced. The fourth section consisted of a list of interview goals formulated as statements (see Table 2) adapted from a previous police survey. The respondents were asked to indicate to what extent they agreed or disagreed with each of the goals. The scale ranged from (1) *Do not agree at all* to (5) *Completely agree*. The order in which the different goals were presented was reverse counterbalanced.

**Table 1:** List of different interview tactics.

**Tactic**	**Survey statement**
Treat friendly	You treat the suspect in a friendly way
Raise voice	You raise your voice during the conversation with the suspect
Care about feelings	You show that you care about the suspects feelings
Interrupt	You interrupt the suspect while he/she is talking
Common interests	You try to find common interests to talk to the suspect about
Boss	You show who's boss
Show respect	You show respect for the suspect as a human being
Confront with info	You confront the suspect with information about the case
Emphasize seriousness	You put pressure by emphasizing the seriousness of the situation
Let suspect talk	You let the suspect finish talking even if he/she talks about irrelevant details
Active listening	You actively listen to what the suspect says
Show what you think	You show the suspect what you think about the criminal act
Unanticipated questions	You ask unanticipated questions
Claim suspect has more info	You claim to know that the suspect has more information then he/she tells you

*Statements as presented in the survey. Participants indicated how often they used each tactic on a Likert scale ranging from Never (1) to Always (5)*.

**Table 2:** List of different interview goals.

**Goals**	**Survey statement**
Confession	Receiving a confession from the suspect is a high priority
Full report	Receiving the suspect's full report is a high priority
Unknown information	Receiving previously unknown information during an interrogation is a high priority
Explanation	Receiving the suspect's explanations regarding existing investigative information is a high priority

*Statements as presented in the survey. Participants indicated to what extent they agreed to each goal on a Likert scale ranging from Do not agree (1) to Completely agree (5)*.

In the fifth section, the respondents were asked to assess how often they found themselves in emotionally demanding situations during their work with interviewing CSA suspects (1 = *Never*, 5 = *Always*), and to what extent their work with interviewing CSA suspects is emotionally demanding (1 = *To a very low extent*, 5 = *To a very high extent*). The respondents were also asked to describe how often they experienced a range of different emotions during their interviews with CSA suspects. The scale was adapted from Ask and Pina (2011), and the scale step for each item ranged from (1) *Never*, to (5) *Always*. The order in which the emotions were presented was reverse counterbalanced, with one-half of the participants rating the emotions in reversed order. Lastly, the respondents were asked an open-ended question about how they manage different emotions that may arise inside them during a suspect interview in CSA cases.

The survey was created in Swedish and translated to Norwegian using forward and back-translations. One of the authors, a native Norwegian researcher fluent in Swedish, initially translated the survey in consultation with another researcher fluent in both Norwegian and Swedish. After the survey was translated, a third researcher fluent in both languages reviewed the translations, and all three agreed upon the final version of the translation. The Norwegian survey was thereafter translated back to Swedish by a native Swedish speaker fluent in Norwegian. Some minor changes were made during this stage of the process. Both versions of the survey were pre-tested prior to data collection for clarity and terminology. The demographic questions regarding training were based on the course curriculums offered in each country.

### Procedure

#### Design

We used a QUAN+qual embedded mixed-methods design (Creswell and Clark, 2017) in which quantitative and qualitative data answered different research questions, were collected at the same occasion, analyzed separately, and then merged. In this study, quantitative data (e.g., Likert scale responses) from the survey were used to investigate Swedish and Norwegian police interviewers' goals, tactics and emotions, and the potential relationship between variables. The qualitative data (open-ended responses) from the survey were used to explore how police interviewers manage emotions that arise during suspect interviews.

#### Data Collection

Verbal information and a pen-and-paper version of the survey was presented to approximately 160 Swedish practitioners during a national conference for specialized child interviewers in March 2019. The same recruitment method was used during a Norwegian national conference in October 2019 for approximately 100 specialized child interviewers. However, unlike in Sweden, the majority of the Norwegian child interviewers did not carry out interviews with CSA suspects. Due to this unforeseen circumstance, we carried out a second data collection with Norwegian practitioners between March and June 2020. For practical reasons following the coronavirus outbreak, we used an online version of the survey. Invitations to participate and a link to the survey was sent out to five large police districts across Norway. The survey was also distributed on social media via Linked-In, Twitter, and Facebook. The participants did not receive any compensation for taking part in the survey.

### Analyses

#### Quantitative Analyses

For the quantitative analyses, we combined the data from the Swedish and Norwegian samples (*N* = 178). We analyzed police interviewers' responses to the quantitative items primarily using bivariate correlations, to examine the relationships between goals, tactics, and emotions. Using the emotion adjectives for which participants provided endorsement ratings, we created five composites based on the conceptual groupings of the emotion words: *anger* (angry, mad; α = 0.81; all reliability coefficients calculated using polychoric correlations), *disgust* (disgusted, sickened; α = 0.66), *interest* (engaged, interested; α = 0.76), *fear* (nervous, scared, worried; α = 0.82), *sadness* (sad, sorrowful; α = 0.60), and *frustration* (frustrated, irritated; α = 0.62). Additionally, we examined the extent to which interviewers from the Swedish and Norwegian samples differed in terms of their self-reported goals, tactics, and emotions by calculating standardized mean differences (*d*) between the samples.

#### Qualitative Analyses

For the qualitative analyses, we combined the Swedish (*N* = 120) and Norwiegan (*N* = 33) police interviewers' responses to the open-ended question about strategies for managing emotions during CSA suspect interviews. The responses were analyzed using data-driven thematic analysis (Braun and Clarke, 2006). Initially, two of the authors separately created code labels that closely matched the content of the written responses. All codes were cross-compared and merged to a thematic structure including themes and sub-themes. Disagreements were resolved through discussion, and the data was re-coded into the final thematic structure. To assess the inter-rater reliability of the coding system, a research assistant coded 20% of the data following the thematic structure. The inter-rater reliability, estimated using Gwet's AC1 for each subtheme, showed very good agreement (*M* = 0.954, *range* = 0.936–0.964). Lastly, quotations to illustrate the qualitative process were selected, edited to facilitate reading, and translated to English.

## Results

### Quantitative Analysis: Self-Reported Interview Goals, Tactics, and Emotions

#### Interview Goals

Swedish police interviewers reported prioritizing obtaining a confession (*M* = 2.61, *SD* = 1.11) to a greater extent than Norwegian interviewers (*M* = 2.09, *SD* = 0.98), *d* = 0.47 [0.14, 0.79]. Interviewers in the Swedish (*M* = 4.50, *SD* = 0.76) and Norwegian (*M* = 4.69, *SD* = 0.47) samples reported prioritizing to a similar extent getting a complete account from the suspect, *d* = −0.28 [−0.60, 0.05]. Norwegian interviewers reported prioritizing obtaining previously unknown information (*M* = 4.58, *SD* = 0.64) to a greater extent than Swedish interviewers (*M* = 4.06, *SD* = 0.97), *d* = −0.56 [−0.89, −0.23]. Swedish interviewers reported prioritizing getting an explanation for existing investigative information (*M* = 4.60, *SD* = 0.72) to a greater extent than Norwegian interviewers (*M* = 4.21, *SD* = 0.82), *d* = 0.49 [0.17, 0.82].

#### Interview Tactics

Table 3 displays descriptive statistics for each sample's self-reported interview tactics, as well as standardized effect sizes estimating the differences between each sample for each tactic. Both the Swedish and Norwegian samples reported using nearly all of the listed tactics with some regularity. All tactics were reported with mean and median frequencies of use above the lower endpoint of the scale (i.e., 1), with the exception of “showing the suspect what you think about the crime” (*Mdn* = 1).

**Table 3:** Descriptive statistics and sample differences for reported interview tactic usage.

	**Sweden**				**Norway**				
**Strategy**	***M***	***SD***	***Mdn***	***Range***	***M***	***SD***	***Mdn***	***Range***	***d[95% CI]***
Treat friendly	4.83	0.37	5	4, 5	5.00	0.00	5	5, 5	−0.50 [−0.82, −0.17]
Raise voice	1.95	0.63	2	1, 3	1.55	0.54	2	1, 3	0.64 [0.31, 0.97]
Care about feelings	3.86	0.86	4	2, 5	3.90	0.63	4	3, 5	−0.05 [−0.38, 0.27]
Interrupt	2.16	0.54	2	1, 4	2.12	0.38	2	1, 3	0.09 [−0.24, 0.41]
Common interests	2.58	1.13	3	1, 5	3.35	1.14	3	1, 5	−0.65 [−0.97, −0.32]
Boss	3.22	1.10	3	1, 5	3.28	1.07	3	1, 5	−0.05 [−0.38, 0.27]
Show respect	4.89	0.44	5	1, 5	5.00	0.00	5	5, 5	−0.30 [−0.62, 0.03]
Confront with info	4.10	0.81	4	1, 5	4.35	0.62	4	3, 5	−0.32 [−0.65, 0.00]
Emphasize seriousness	2.98	0.86	3	1, 5	2.42	0.89	2	1, 4	0.62 [0.29, 0.94]
Let suspect talk	3.68	0.70	4	1, 5	3.46	0.64	3	2, 5	0.32 [0.00, 0.65]
Active listening	4.88	0.33	5	4, 5	4.85	0.36	5	4, 5	0.10 [−0.22, 0.43]
Show what you think	1.37	0.58	1	1, 3	1.46	0.58	1	1, 3	−0.15 [−0.48, 0.17]
Unanticipated questions	3.15	0.75	3	1, 5	3.06	0.72	3	1, 4	0.12 [−0.21, 0.44]
Claim suspect has more info	2.10	0.92	2	1, 5	2.77	1.62	2	1, 5	−0.56 [−0.89, −0.23]

*n = 126 for Sweden and n = 52 for Norway. M = mean, SD = standard deviation, Mdn = median, d[95% CI] = standardized mean difference with 95% confidence interval*.

Some notable differences between the Swedish and Norwegian samples were evident. For example, Swedish interviewers reported raising their voice, *d* = 0.64 [0.31, 0.97], and emphasizing the seriousness of the offense, *d* = 0.62 [0.29, 0.94], more frequently than Norwegian interviewers, and Swedish interviewers reported approaching the suspect in a friendly manner less frequently than Norwegian interviewers, *d* = −0.50 [−0.82, −0.17]. Norwegian interviewers also reported finding common interests with the suspect, *d* = −0.65 [−0.97, −0.32], and saying that the suspect had more information than they were providing, *d* = −0.56 [−0.89, −0.23], more frequently than Swedish interviewers.

#### Emotions

Table 4 displays descriptive statistics for each sample's self-reported emotions, as well as standardized effect sizes estimating the differences between each sample for each emotion composite. Both the Swedish and Norwegian samples reported experiencing nearly all the listed emotions with some regularity. All emotion composites had means and medians above the lower endpoint of the scale. Swedish interviewers reported to more frequently feel interested, *d* = 0.44 [0.11, 0.77], and disgusted, *d* = 0.54 [0.22, 0.87], during suspect interviews, compared to Norwegian interviewers.

**Table 4:** Descriptive statistics and sample differences for reported experienced emotions.

	**Sweden**				**Norway**				
	***M***	***SD***	***Mdn***	***Range***	***M***	***SD***	***Mdn***	***Range***	***d* [95% CI]**
Anger	2.23	0.74	2.00	1.00, 4.00	2.03	0.75	2.00	1.00, 3.50	0.26 [−0.07, 0.58]
Disgust	2.27	0.81	2.00	1.00, 4.50	1.86	0.53	2.00	1.00, 3.00	0.54 [0.22, 0.87]
Sadness	2.20	0.76	2.00	1.00, 4.00	2.09	0.80	2.00	1.00, 4.00	0.14 [−0.19, 0.47]
Fear	1.75	0.56	1.67	1.00, 4.00	1.80	0.61	1.67	1.00, 3.00	−0.09 [−0.42, 0.23]
Interest	4.38	0.60	4.50	1.00, 5.00	4.11	0.61	4.00	2.00, 5.00	0.44 [0.11, 0.77]
Frustration	2.75	0.53	3.00	1.00, 4.00	2.68	0.66	3.00	1.00, 4.50	0.11 [−0.21, 0.44]

*n = 126 for Sweden and n = 52 for Norway. M = mean, SD = standard deviation, Mdn = median, d [95% CI] = standardized mean difference with 95% confidence interval*.

Overall, participants on average reported that they experienced emotionally demanding situations in their work rarely or sometimes (*M* = 2.62, *SD* = 0.80). Additionally, Swedish police interviewers (*M* = 2.73, *SD* = 0.77) reported being more frequently in emotionally demanding situations compared to Norwegian interviewers (*M* = 2.37, *SD* = 0.82), *d* = 0.45 [0.12, 0.78]. On average, participants reported that they found their work emotionally demanding sometimes (*M* = 3.08, *SD* = 1.05). However, Swedish police interviewers (*M* = 2.94, *SD* = 0.98) reported that their work is emotionally demanding to a lesser extent compared to Norwegian interviewers (*M* = 3.40, *SD* = 1.14), *d* = −0.43 [−0.76, −0.11].

#### Correlations Between Goals, Tactics, and Emotions

Table 5 displays the correlations between interview goals and self-reported use of tactics. Several tactics have a significant correlation with the four goals. The goal of obtaining a confession was significantly positively correlated with raising one's voice, emphasizing the seriousness of the offense, showing the suspect what you think about the crime, and claiming that the suspect has more information. The goal of obtaining a full report was significantly positively correlated with confronting the suspect with information and engaging in active listening and significantly negatively correlated with interrupting the suspect. The goal of obtaining previously unknown information was significantly positively correlated with finding common interests with the suspect, confronting the suspect with information, and with claiming the suspect has more information. The goal of obtaining an explanation for the offense was significantly positively correlated with showing the suspect who is boss and confronting the suspect with information.

**Table 5:** Bivariate correlations and 95% confidence intervals of interview tactics and goals.

	**Confession**	**Full report**	**Unknown information**	**Explanation**
Treat friendly	−0.038 [−0.184, 0.109]	0.103 [−0.045, 0.246]	−0.018 [−0.164, 0.129]	−0.115 [−0.258, 0.032]
Raise voice	**0.173 [0.027, 0.312]**	−0.062 [−0.207, 0.085]	−0.140 [−0.281, 0.006]	0.011 [−0.136, 0.157]
Care about feelings	−0.023 [−0.169, 0.124]	0.126 [−0.021, 0.268]	0.030 [−0.117, 0.176]	0.073 [−0.075, 0.217]
Interrupt	0.042 [−0.106, 0.187]	**–0.154 [–0.294**, –**0.008]**	−0.010 [−0.156, 0.137]	0.008 [−0.139, 0.154]
Common interests	−0.043 [−0.189, 0.104]	0.045 [−0.103, 0.190]	**0.170 [0.024, 0.309]**	−0.037 [−0.183, 0.110]
Boss	0.136 [−0.011, 0.277]	0.034 [−0.114, 0.179]	0.032 [−0.115, 0.178]	**0.158 [0.012, 0.298]**
Show respect	−0.022 [−0.168, 0.125]	0.105 [−0.043, 0.248]	0.033 [−0.114, 0.179]	−0.024 [−0.170, 0.123]
Confront with info	0.075 [−0.073, 0.219]	**0.156 [0.010, 0.296]**	**0.170 [0.024, 0.309]**	**0.265 [0.123, 0.396]**
Emphasize seriousness	**0.328 [0.191, 0.453]**	−0.058 [−0.203, 0.089]	0.058 [−0.089, 0.203]	0.102 [−0.045, 0.245]
Let suspect talk	0.023 [−0.124, 0.170]	0.117 [−0.030, 0.259]	−0.013 [−0.159, 0.134]	0.041 [−0.107, 0.186]
Active listening	−0.023 [−0.169, 0.125]	**0.166 [0.019, 0.305]**	−0.075 [−0.219, 0.072]	−0.019 [−0.165, 0.128]
Show what you think	**0.166 [0.020, 0.305]**	−0.092 [−0.236, 0.055]	0.084 [−0.063, 0.228]	0.021 [−0.126, 0.168]
Unanticipated questions	0.114 [−0.034, 0.256]	0.026 [−0.121, 0.172]	−0.015 [−0.161, 0.132]	0.009 [−0.138, 0.155]
Claim suspect has more info	**0.164 [0.017, 0.303]**	0.103 [−0.045, 0.246]	**0.268 [0.126, 0.399]**	0.066 [−0.082, 0.210]

*Correlations whose 95% CI excludes 0 are displayed in boldface*.

Table 6 displays the correlations between the six emotion composites and four self-reported goals for suspect interviews. As can be seen, self-reported experiences of anger and frustration were significantly positively correlated with the goal of obtaining a confession. Additionally, experiences of disgust were negatively correlated with the goal of obtaining previously unknown information, and experiences of interest were positively correlated with the goal of obtaining an explanation for the offense.

**Table 6:** Bivariate correlations and 95% confidence intervals of emotion composites and interview goals.

	**Anger**	**Disgust**	**Sadness**	**Fear**	**Interest**	**Frustration**
Confession	**0.231 [0.087, 0.365]**	0.128 [−0.019, 0.270]	0.095 [−0.053, 0.238]	−0.091 [−0.235, 0.056]	0.075 [−0.072, 0.220]	**0.171 [0.025, 0.310]**
Full report	0.058 [−0.089, 0.203]	−0.084 [−0.228, 0.063]	0.033 [−0.114, 0.179]	0.080 [−0.068, 0.224]	0.098 [−0.049, 0.242]	0.100 [−0.047, 0.243]
Unknown information	−0.033 [−0.178, 0.115]	–**0.150 [**–**0.291**, –**0.004]**	0.030 [−0.117, 0.176]	0.009 [−0.138, 0.155]	−0.031 [−0.177, 0.116]	−0.002 [−0.149, 0.145]
Explanation	0.079 [−0.069, 0.223]	0.069 [−0.078, 0.214]	0.093 [−0.054, 0.237]	0.019 [−0.128, 0.166]	**0.197 [0.052, 0.334]**	−0.001 [0.148, 0.146]

*Correlations whose 95% CI excludes 0 are displayed in boldface*.

Table 7 displays the correlations between the six emotion composites and the self-reported use of interview tactics. Self-reported experiences of anger were significantly positively correlated with raising one's voice, emphasizing the seriousness of the crime, and asking unanticipated questions, and anger was significantly negatively correlated with expressing care for the suspect's feelings. Experiences of disgust were positively correlated with raising one's voice, emphasizing the seriousness of the crime, asking unanticipated questions, and disgust was negatively correlated with treating the suspect in a friendly manner. Sadness was not significantly correlated with the self-reported use of any interview tactics. Fear was significantly negatively correlated with showing respect for the suspect and with claiming the suspect has more information. Interest was significantly positively correlated with expressing care for the suspect's feelings. Frustration was significantly positively correlated with raising one's voice and with emphasizing the seriousness of the offense.

**Table 7:** Bivariate correlations and 95% confidence intervals of interview tactics and emotion composites.

	**Anger**	**Disgust**	**Sadness**	**Fear**	**Interest**	**Frustration**
Treat friendly	−0.094 [−0.237, 0.054]	**–0.176 [**–**0.315**, –**0.030]**	−0.015 [−0.161, 0.132]	0.017 [−0.130, 0.164]	0.122 [−0.025, 0.264]	−0.014 [−0.160, 0.133]
Raise voice	**0.280 [0.139, 0.410]**	**0.278 [0.137, 0.408]**	−0.025 [−0.171, 0.122]	0.115 [−0.032, 0.258]	−0.097 [−0.240, 0.050]	**0.229 [0.086, 0.364]**
Care about feelings	**–0.159 [**–**0.299**, –**0.013]**	−0.116 [−0.258, 0.031]	0.102 [−0.045, 0.245]	0.137 [−0.010, 0.278]	**0.177 [0.031, 0.316]**	−0.080 [−0.252, 0.038]
Interrupt	0.090 [−0.057, 0.234]	0.095 [−0.053, 0.238]	−0.031 [−0.177, 0.117]	−0.042 [−0.187, 0.105]	−0.109 [−0.252, 0.038]	0.050 [−0.097, 0.196]
Common interests	−0.006 [−0.152, 0.141]	−0.015 [−0.161, 0.132]	0.045 [−0.102, 0.190]	0.090 [−0.057, 0.234]	0.120 [−0.027, 0.262]	−0.075 [−0.220, 0.072]
Boss	0.097 [−0.051, 0.240]	−0.012 [−0.158, 0.135]	−0.020 [−0.166, 0.127]	−0.100 [−0.243, 0.047]	0.061 [−0.086, 0.206]	−0.035 [−0.181, 0.112]
Show respect	−0.062 [−0.207, 0.085]	−0.054 [−0.200, 0.093]	−0.050 [−0.196, 0.097]	–**0.200 [**–**0.337**, –**0.055]**	0.081 [−0.067, 0.225]	−0.008 [−0.155, 0.139]
Confront with info	0.047 [−0.101, 0.192]	0.045 [−0.103, 0.190]	−0.100 [−0.243, 0.048]	−0.067 [−0.211, 0.081]	0.096 [−0.051, 0.240]	−0.022 [−0.168, 0.125]
Emphasize seriousness	**0.323 [0.185, 0.448]**	**0.280 [0.139, 0.410]**	0.141 [−0.006, 0.281]	0.139 [−0.008, 0.280]	−0.060 [−0.205, 0.088]	**0.335 [0.198, 0.459]**
Let suspect talk	0.020 [−0.127, 0.166]	−0.007 [−0.153, 0.140]	0.005 [−0.142, 0.152]	−0.103 [−0.246, 0.044]	0.134 [−0.013, 0.275]	−0.015 [−0.161, 0.132]
Active listening	−0.090 [−0.234, 0.057]	−0.073 [−0.217, 0.074]	−0.055 [−0.200, 0.093]	−0.081 [−0.225, 0.066]	0.076 [−0.072, 0.220]	−0.110 [−0.253, 0.037]
Show what you think	0.068 [−0.080, 0.212]	0.066 [−0.082, 0.211]	−0.029 [−0.175, 0.118]	−0.020 [−0.166, 0.127]	−0.058 [−0.203, 0.089]	0.092 [−0.056, 0.235]
Unanticipated questions	**0.160 [0.014, 0.300]**	**0.197 [0.051, 0.334]**	0.005 [−0.142, 0.151]	−0.057 [−0.202, 0.090]	0.079 [−0.068, 0.223]	0.115 [−0.032, 0.257]
Claim suspect has more info	0.100 [−0.047, 0.244]	0.007 [−0.140, 0.154]	−0.021 [−0.167, 0.126]	–**0.171 [**–**0.310**, –**0.025]**	−0.122 [−0.264, 0.025]	−0.064 [−0.209, 0.083]

*Correlations whose 95% CI excludes 0 are displayed in boldface*.

### Qualitative Analysis: How Police Interviewers Manage Emotions

A total of 120 Swedish and 33 Norwegian police interviewers provided a written response to the open-ended question “How do you manage different emotions that may arise within you in connection to an interview of a CSA suspect?” The thematic analysis revealed that a majority of police interviewers expressed that their emotions did not belong in the interview room, which is captured in the main theme *Putting emotions aside during the interview*. Furthermore, most police interviewers described how they tried to manage their emotions in different ways outside of the interview room, which is captured in the main theme *Coping with emotions afterwards*. Each theme had three subthemes, see Table 8.

**Table 8:** Overview of the thematic structure and the proportion of responses categorized into each sub-theme. (*n* = 153 respondents).

**Theme**	**Sub-theme**	**Responses**
*Putting emotions aside*	Focus on one's professional role	85 (55.6%)
*during the interview*	See the human behind the act	10 (6.5%)
	Take a break	8 (5.2%)
*Coping with*	Share emotions with others	98 (64.1%)
*emotions afterwards*	Take care of oneself	25 (16.3%)
	Reflect on the experience	9 (5.9%)

*The thematic analysis is based on open survey answers from 120 Swedish and 33 Norwegian police interviewers*.

#### Putting Emotions Aside During the Interview

The consensus among respondents seemed to be that the interviewer's emotions should not be expressed during a suspect interview. Some police interviewers described this as an easy task (e.g., “*It is a job I am used to and have learned to handle*”), whereas other practitioners described how they in different ways had to work actively on putting their emotions aside by using different strategies. These strategies could be categorized into three different subthemes: (1) *Focus on one's professional role, (*2) *See the human behind the act*, and (3) *Take a break*.

##### Focus on One's Professional Role

The most frequently mentioned way to manage emotions during the interview was reminding oneself about one's professional role. The answers provided insight into different ways this could be done. Oftentimes, remaining professional involved suppressing one's personal feelings:

You adopt a professional role where your own emotions are put aside. The goal is to get the suspect to talk, to put his/her experiences and knowledge about what happened into words. Show respect—gather the information that is needed to clarify guilt or innocence. My emotions are not expressed during the interview situation (Norwegian police officer, 17 years of experience interviewing CSA suspects).

Other strategies were keeping their guard up and not letting emotions arise in the first place, or not putting any personal judgement on the criminal act. Many interviewers found it helpful to focus on the task in order to avoid being emotional, and some described how it could also be a way of motivating oneself when facing tasks that were more challenging than others:

What can sometimes be difficult is to ask intimate questions related to the sexual parts, or to ask about more details on a specific topic (because you pressure the one being questioned). It is a kind of invasion of another person's private sphere, even though the situation has the framework that it has. I deal with this by convincing myself that it is natural to talk about this, and that the situation requires it. I do what I can to both appear and sound neutral. And sometimes I explain the reason for asking these questions (Norwegian police officer, 5 years of experience interviewing CSA suspects).

Most officers attempted to suppress their feelings, but they also acknowledged that this could be a difficult task. One police officer explained how interviewing suspects of CSA could be especially stressful, particularly in cases where the same police officer had first interviewed the child victim:

It is okay to have emotions, but they must be handled the right way. If I experience too much emotion, I should leave the interview to my colleague. This is especially important if I have conducted a child interview that evoked a lot of feelings (Swedish police officer, 9 years of experience interviewing CSA suspects).

According to some police interviewers, controlling one's emotions was an ability developed by experience and thus got easier over time. A few police interviewers revealed that they occasionally had failed to do so, which could result in them raising their voice against the suspect. These situations were described as a “loss of control” and thus not a planned strategy. Contrasting this common belief that emotions should not be expressed, a few argued that showing emotions could be a way of highlighting the seriousness of the situation. One police officer explained that it was not the initial strategy, but something one might have to resort to in exceptional cases:

I enter the room with an attitude of not knowing what has happened. I have received a story from a child and now I want to know the suspect's story. If the suspect does not take the child's story seriously, I might emphasize the gravity of the situation (Swedish police officer, 6 years of experience interviewing CSA suspects).

##### See the Human Behind the Act

Building a positive, open, and respectful environment was described as a way of gaining as much information as possible during the interview. This could be accomplished by trying to feel empathy for the suspect as a human being, without neglecting the seriousness of the crime that the suspect was accused of committing.

Some emotions can be good, like feeling sympathy for the person behind the crime. /…/ The slightest sense on their part that you feel anger, contempt etc., means that you will be met with the same, and you will not get any answers. It is possible to get answers to very intimate questions and to push on specific questions if the person is treated with respect and without judgment (Swedish police officer, 10 years of experience interviewing CSA suspects).

In some cases, it could be difficult to feel empathy for the suspect. The answers revealed how it could be useful to try focusing on the suspect as a multifaceted person, with both good and bad sides, and not being judgmental.

It helps me to think “he did not know better,” “his home life is bad,” “lack of upbringing,” to have an explanation to myself (which I of course do not say). It is difficult when you want them to feel guilt (if they do not express it) and at the same time not make them feel bad (they are probably already feeling bad, plus it is not our job to handle such a thing) (Swedish police officer, 1 year of experience interviewing CSA suspects).

##### Take a Break

If negative emotions did arise during the interview, several police interviewers described how they tried to regain their focus by pausing shortly. Some mentioned how a break could relieve problematic emotions before continuing the interview: “If I feel frustrated I take a break and talk to my colleague in the back room, or a break with fresh air for us both” (Norwegian police officer, 6 years of experience interviewing CSA suspects). Others described how they could make sure to get a mental break in the middle of the interview by taking a sip of water, taking a deep breath, flipping through some papers or asking the prosecutor if he/she has any questions: “Note the feeling and ‘let it pass'. Breathe. Take a break.” (Swedish police officer, 7 years of experience interviewing CSA suspects).

#### Coping With Emotions Afterwards

Interviewing suspected CSA offenders could evoke a range of emotions. However, as discussed in the previous main theme, most practitioners agreed that these emotions should not influence their interview procedures. Furthermore, some described that they rarely experienced any strong emotions during the interview, but instead experienced that negative emotions could sometimes appear afterwards. The police interviewers discussed different methods for coping with emotions after leaving the interview room. These strategies could be categorized into three different sub-themes: (1) *Share emotions with others, (*2) *Take care of oneself*, and (3) *Reflect on the experience*.

##### Share Emotions With Others

The most frequently reported strategy for managing emotions was to share their feelings afterwards. Many police interviewers found support among their co-workers. Oftentimes, the police interviewers could vent to their closest colleagues who were knowledgeable about the situation. Since their co-workers shared similar experiences, this form of collegial support was described as very important for their emotion management.

I can talk to my co-workers after the interview about how I felt and how I feel now. I experience that it helps to talk about the emotions that arise. All my co-workers have a great understanding of the emotions that can be present. No one needs to feel stupid, or like it is wrong or something like that (Swedish civilian officer, less than 1 year of experience interviewing CSA suspects).

The Swedish police interviewers also talked about the value of attending individual or group supervision sessions (typically led by a psychologist). This form of regular supervision was described in positive terms. However, some practitioners described that they did not have access to it on a regular basis. In contrast, the Norwegian police interviewers did not mention regular supervision as a strategy for managing emotions. Lastly, a few police interviewers reported that they sometimes shared their feelings with their partner or close friends. This could be challenging, however, as the police interviewers had to leave out case-specific information for sensitivity and confidentiality reasons.

##### Take Care of Oneself

Another commonly reported strategy for managing emotions was to take care of their well-being, both mentally and physically. Many police interviewers stated that they went for a run or did other physical exercises after the interview to clear their mind and release any potential negative emotions. A few described other forms of physical activity, including going on walks outside in the fresh air. Some mentioned the importance of sleep for their mental well-being. Having hobbies and a rich private life was also described as a protective factor for managing emotions that can arise during their line of work.

Training and physical activity outside works if you feel it in your body. Being together with friends and family and keeping yourself busy with other things (Norwegian police officer, 10 years of experience interviewing CSA suspects).

One police officer highlighted that although physical activity or distractions was helpful, it did not replace the need for directly addressing the emotions.

“Exercising, doing completely other stuff, seeing the good world. This is, however, a kind of escape, and it is important to discuss the emotions in the group first.” (Swedish police officer, 5 years of experience interviewing CSA suspects).

##### Reflect on the Experience

Lastly, some police interviewers discussed the importance of reflecting upon the interview process and their performance. This could include self-reflection on how to improve for the next interview.

I always watch the video-recording of the interview and reflect on how it felt, and about my performance. I think this creates some distance to the case and my feelings, and I can learn from any potential mistakes (Swedish police officer, 5 years of experience interviewing CSA suspects).

Others emphasized the importance of reflecting on their own reactions, in order to understand them and learn from their experiences until the next case: “I reflect on my own emotions. Evaluate why they arose and what it triggered in me as a person.” (Swedish civilian officer, 5 years of experience interviewing CSA suspects).

## Discussion

The present study sought to examine police interviewers' self-reported goals, tactics, and emotional experiences when questioning CSA suspects in a Scandinavian context. The Swedish and Norwegian interviewers reported largely similar profiles of emotional experiences when conducting these interviews. Reassuringly, interviewers appear to feel predominantly interested. This was the only feeling that, on average, occurs “often” according to the respondents' self-ratings. Negative emotions typically occur only “sometimes” or even “rarely.” Of these, feelings of frustration seem to be the most prevalent. Although the exact cause of frustration is not apparent from these ratings, they do imply that a failure to meet one's objective is not an uncommon experience when interviewing CSA suspects. Fear-related emotions, like feeling worried and scared, were the least frequently reported, suggesting that interviewers rarely experience a complete lack of control or a sense of threat in the interview room.

Two notable differences were observed between Swedish and Norwegian interviewers' reported emotions: Swedish respondents reported experiencing disgust and interest more frequently than their Norwegian counterparts. This might reflect a higher degree of personal involvement in CSA cases among Swedish interviewers. This interpretation would be consistent with one of the systemic differences between the countries: In Sweden, the same interviewer is often involved in both the child victim and suspect interview. In contrast, Norwegian child interviewers often try to avoid interviewing the suspect in the same case (Detective superintendent Inger-Lise Brøste, personal communication, 30 August 2019). This difference became evident during data collection, where many Norwegian child interviewers did not meet the inclusion criterion of having experience conducting suspect interviews during the last 5 years. It seems plausible that a more extensive personal involvement in a case would generate more intense emotional reactions. This explanation is speculative, but it raises the interesting possibility that reducing personal involvement may be an effective measure to minimize unwanted emotional interference in criminal investigations.

The observed correlations between emotions and interviewing goals and tactics were typically weak. This is perhaps to be expected considering that respondents were asked to report generalities across several cases rather than reflect on any particular case. Nevertheless, we were able to detect a few patterns that deserve attention: First, the frequency of experiencing anger was positively associated with the goal of obtaining a confession and with aggressive tactics like raising one's voice, emphasizing the seriousness of the crime, and not caring about the suspect's feelings. From an emotion-theoretical perspective, these correlations make sense. The experience of anger is associated with action tendencies that facilitate confrontational and confirmation-seeking behavior (Lerner and Tiedens, 2006; Carver and Harmon-Jones, 2009). Second, the reported frequency of experiencing frustration displayed a similar pattern as anger, which may indicate that some interviewers resort to aggressive tactics when facing frustrating obstacles in the interview room. Third, disgust was similarly associated with aggressive tactics and negatively, albeit weakly, associated with the goal of obtaining previously unknown information. The fact that disgust is associated with avoidant action tendencies (Frijda et al., 1989) might explain why the emotion correlates with a reduced interest in further information. In addition, feelings of disgust are intimately associated with the act of moral condemnation (Chapman and Anderson, 2013), which may explain the link between the emotion and use of aggressive interview tactics.

It is important to note that our correlational data cannot distinguish between the different causal mechanisms that may produce the observed relationships between emotions and interview goals and tactics. For instance, the possibility that emotions precede the employment of goals and tactics (e.g., anger increases the motivation to obtain a confession) is equally plausible as the opposite causal direction (e.g., confession-seeking behavior produces frustrating suspect responses). Moreover, the correlations might be due to unspecified individual differences among interviewers (e.g., personality characteristics may influence both the likelihood of experiencing anger and the preference for aggressive interview tactics). This limitation notwithstanding, our correlational findings highlight the relevance of emotional processes in CSA suspect interrogations and raise the need for further exploration of the topic.

Despite the quantitative results indicating that emotions do play a role in CSA suspect interviews, the participants asserted in their open-ended responses that emotions should not and do not affect their work. The qualitative analysis showed strong consensus regarding the importance of not expressing emotions during the suspect interview, or letting emotions influence their interview procedures. To achieve these aims, the police interviewers described several strategies for managing their emotional reactions and coping with emotions after the suspect interview. It is possible that a similar thematic structure would be applicable if asking police interviewers about managing emotions in investigations of other violent crimes. However, cases involving children are often perceived as especially distressing (Brown et al., 1999; Oxburgh et al., 2015; Huey and Kalyal, 2017). A few officers explicitly mentioned some unique aspects of CSA investigations that could be particularly challenging, for instance, having to ask intimate questions of a sexual nature. While this could be perceived as invading the private sphere of the suspect, it is a crucial task in a CSA investigation, illustrating the high demand put on the professionals.

In line with previous research emphasizing the importance of emotional support from co-workers as a coping aid (Burns et al., 2008; Powell et al., 2014), both Swedish and Norwegian officers frequently expressed that sharing emotions with colleagues was helpful. Interestingly, Swedish police officers also commonly mentioned the benefits of attending regular psychology sessions provided by their work, which was not discussed by the Norwegian officers. In previous studies, the attitudes toward receiving psychological support have varied, with the main reason for dissatisfaction being that psychologists lack specialized knowledge about investigating sensitive cases and thus came across as uncomfortable talking about specific topics (Powell et al., 2014). A fear of the possible repercussions of utilizing psychological support (e.g., that it is considered to be something negative if applying for promotion), and a stigma associated with officers seeking help has also been reported (Cullen et al., 2020; Foley et al., 2021). Furthermore, having understanding and knowledgeable supervisors is also important (Powell et al., 2014), as inadequate organizational support has been reported as a factor underlying burnout (Fansher et al., 2020).

Another aspect highlighted in the open-ended responses, was that the interview with the child victim might evoke many emotions, which was described as a complicating factor when trying to remain neutral in the subsequent interview with the suspect. While this contrasts previous findings that police interviewers used fewer negative emotional utterances during the interview of a CSA suspect if they had first interviewed the child (Oxburgh et al., 2006), it is possible that police interviewers manage to remain neutral, but that it is highly emotionally taxing for them.

The qualitative analysis also provided some insight into some police interviewers' goals of the suspect interview. Several officers described how their main task was to get the suspect to talk and provide his or her view on the allegations, while a few described how they emphasized the seriousness of the crime in order to evoke feelings of guilt in the suspect. Thus, based on the open-ended responses, information-gathering tactics were much more common than accusatory techniques. Similarly, the quantitative results indicate that information-gathering goals (e.g., collecting previously unknown information and getting explanations for investigative information) were prioritized to a greater extent than obtaining a confession. Moreover, interview tactics focused on collecting information and building rapport (e.g., active listening, showing respect, and letting the suspect talk) were among those reported to be used most frequently, consistent with observational research examining the tactics used in countries that have implemented investigative interviewing approaches (see, e.g., Soukara et al., 2009).

Although the police organizations in Sweden and Norway share some similarities, there are significant systematic differences that could help shed light on the variations in tactics and goals across countries. With the implementation of KREATIV, the shift toward a research-based interview model occurred nearly two decades ago in Norway. While Sweden is currently working toward implementing a national model, it will take time before the entire police force has received training in the technique. The lack of a research-based interview model may help explain some of the differences observed between the two countries. For example, we observed that Swedish interviewers reported more frequently using confrontational tactics, such as raising one's voice and emphasizing the seriousness of the crime, whereas Norwegian interviewers reported using these tactics less. Swedish interviewers also reported prioritizing obtaining confessions to a higher degree than their Norwegian counterparts. These differences may be due, in part, to the KREATIV model emphasizing gathering information rather than seeking a confession (Fahsing et al., 2016). As Sweden continues to work toward implementing its new interview model, these differences may diminish over time.

Furthermore, both countries are currently lacking specialized interview guidelines focused on investigations involving alleged CSA. Given the nature of these cases (e.g., the scarcity of corroborative evidence: Ernberg et al., 2018), the development of research-based interview guidelines adapted for suspected CSA offenders may facilitate the police in their daily work. Indeed, research-based guidelines for CSA cases are especially important, given the quality of currently available practical recommendations. For example, the Reid Technique—an accusatory interrogation approach widely criticized by researchers and legal scholars (see, e.g., Kassin et al., 2010)—offers a manual on interrogations in CSA cases (Buckley, 2015), closely based on its more general system of interrogating suspects (Inbau et al., 2013), which advocates the use of problematic tactics (e.g., minimization; Luke and Alceste, 2020).

## Limitations

Some methodological limitations of the current research need to be addressed. First, the findings are correlational, and we can therefore not draw any conclusions regarding causality or directionality. Second, the data are based on retrospective self-reports from practitioners. Survey data can be affected by a range of response biases, including social desirability effects (Tourangeau et al., 2000). Furthermore, we asked the participants about their general perceptions and experiences of interviewing CSA suspects. Their goals, tactics, and emotional experiences might differ between interviews depending on the situation at hand (e.g., the amount of corroborative evidence available, whether the suspect is cooperative or uncooperative). Due to limitations with self-reports, systematic field research is needed to study the techniques used by Swedish and Norwegian practitioners inside the interview rooms. Finally, our sample was relatively small (total *N* = 176), and as a consequence, our ability to estimate small and subtle differences is limited. The small sample is especially concerning given that the correlations we observed between goal, tactics, and emotions were small in size. That being said, given that there are relatively few police interviewers in Sweden and Norway who conduct interviews with suspects of CSA, our sample nevertheless represents a substantial portion of the total population.

## Conclusions

This study set out to explore Swedish and Norwegian police interviewers' goals, tactics, and emotional experiences when questioning suspected CSA offenders. The quantitative data showed correlations between officers' self-reported emotional experience and the tactics they use and the goals they prioritize, suggesting that emotions play a role in the interview of suspected CSA offenders. In the qualitative analysis, however, participants asserted that emotions should not and do not affect their work. They described a range of different strategies that could be used to put emotions aside during the interview and instead manage them afterwards. Given the low prosecution rates and limited access to corroborative evidence in CSA investigations (Ernberg et al., 2018), more research is needed to better understand how to effectively interview CSA suspects. The potential link between interviewers' goals, tactics, and emotional experiences should be kept in mind during the development of such guidelines.

## Data Availability Statement

The quantitative data supporting the conclusions of this article will be made available in aggregated form by the authors on request, without undue reservation.

## Ethics Statement

Ethical review and approval was not required for the study on human participants in accordance with the local legislation and institutional requirements. The patients/participants provided their written informed consent to participate in this study.

## Author Contributions

MM, MJ, and TL designed the study. MM, MJ, and MLS collected the data. MM and MJ carried out the qualitative analyses. TL and KA carried out the quantitative analyses. All authors discussed the results and worked on the manuscript.

## Conflict of Interest

The authors declare that the research was conducted in the absence of any commercial or financial relationships that could be construed as a potential conflict of interest.
