# Supplementary material for: Swedish and Norwegian Police Interviewers' Goals, Tactics, and Emotions When Interviewing Suspects of Child Sexual Abuse
Source: Front Psychol. 2021 Jul 9;12:606774. doi: 10.3389/fpsyg.2021.606774 (PMC8298905; doi:10.3389/fpsyg.2021.606774)
Supplement: Supplementary file 1 [file Data_Sheet_1.ZIP › EFA_norway.html]

Factor analysis of reported strategies and reported emotions – Norwegian sample


# Factor analysis of reported strategies and reported emotions – Norwegian sample

# Imputations for factor analysis

```
set.seed(123)
```

```
strategies <- data %>% 
  select(starts_with("strategies"))

emotions <- data %>% 
  select(starts_with("emotions"))
```

```
strat.impute <- mice(strategies, m = 5, method = "pmm")
```

```
## 
##  iter imp variable
##   1   1  strategies_2  strategies_6  strategies_13
##   1   2  strategies_2  strategies_6  strategies_13
##   1   3  strategies_2  strategies_6  strategies_13
##   1   4  strategies_2  strategies_6  strategies_13
##   1   5  strategies_2  strategies_6  strategies_13
##   2   1  strategies_2  strategies_6  strategies_13
##   2   2  strategies_2  strategies_6  strategies_13
##   2   3  strategies_2  strategies_6  strategies_13
##   2   4  strategies_2  strategies_6  strategies_13
##   2   5  strategies_2  strategies_6  strategies_13
##   3   1  strategies_2  strategies_6  strategies_13
##   3   2  strategies_2  strategies_6  strategies_13
##   3   3  strategies_2  strategies_6  strategies_13
##   3   4  strategies_2  strategies_6  strategies_13
##   3   5  strategies_2  strategies_6  strategies_13
##   4   1  strategies_2  strategies_6  strategies_13
##   4   2  strategies_2  strategies_6  strategies_13
##   4   3  strategies_2  strategies_6  strategies_13
##   4   4  strategies_2  strategies_6  strategies_13
##   4   5  strategies_2  strategies_6  strategies_13
##   5   1  strategies_2  strategies_6  strategies_13
##   5   2  strategies_2  strategies_6  strategies_13
##   5   3  strategies_2  strategies_6  strategies_13
##   5   4  strategies_2  strategies_6  strategies_13
##   5   5  strategies_2  strategies_6  strategies_13
```

```
## Warning: Number of logged events: 2
```

```
strat.comp <- complete(strat.impute)

strat.comp.fa <- strat.comp %>% 
  select(-strategies_1, -strategies_7)
```

```
emot.impute <- mice(emotions, m = 5, method = "pmm")
```

```
## 
##  iter imp variable
##   1   1  emotions_5  emotions_14
##   1   2  emotions_5  emotions_14
##   1   3  emotions_5  emotions_14
##   1   4  emotions_5  emotions_14
##   1   5  emotions_5  emotions_14
##   2   1  emotions_5  emotions_14
##   2   2  emotions_5  emotions_14
##   2   3  emotions_5  emotions_14
##   2   4  emotions_5  emotions_14
##   2   5  emotions_5  emotions_14
##   3   1  emotions_5  emotions_14
##   3   2  emotions_5  emotions_14
##   3   3  emotions_5  emotions_14
##   3   4  emotions_5  emotions_14
##   3   5  emotions_5  emotions_14
##   4   1  emotions_5  emotions_14
##   4   2  emotions_5  emotions_14
##   4   3  emotions_5  emotions_14
##   4   4  emotions_5  emotions_14
##   4   5  emotions_5  emotions_14
##   5   1  emotions_5  emotions_14
##   5   2  emotions_5  emotions_14
##   5   3  emotions_5  emotions_14
##   5   4  emotions_5  emotions_14
##   5   5  emotions_5  emotions_14
```

```
emot.comp <- complete(emot.impute)

emot.comp.fa <- emot.comp
```

# Factor analysis

## Strategies

```
cor(strat.comp.fa)
```

```
##               strategies_2 strategies_3 strategies_4 strategies_5 strategies_6
## strategies_2    1.00000000   0.15385619  0.265179430  -0.11785752   0.21502879
## strategies_3    0.15385619   1.00000000  0.047113704   0.45543247   0.27396107
## strategies_4    0.26517943   0.04711370  1.000000000  -0.04912281   0.29617444
## strategies_5   -0.11785752   0.45543247 -0.049122807   1.00000000   0.11970384
## strategies_6    0.21502879   0.27396107  0.296174439   0.11970384   1.00000000
## strategies_8    0.07613450  -0.11266869 -0.006399877  -0.28372789  -0.01575618
## strategies_9    0.16856409  -0.09982774 -0.031225428  -0.08921551   0.37182612
## strategies_10   0.11753804   0.15963635 -0.062209075   0.23432085  -0.15315563
## strategies_11  -0.16837611   0.27412948 -0.153117524  -0.24790456   0.02307965
## strategies_12   0.06776662  -0.03715172  0.020751434  -0.00922286   0.17759507
## strategies_13  -0.09828519  -0.15366614 -0.083230668   0.16472736  -0.01463641
## strategies_14  -0.19149091   0.01618328 -0.019722149   0.21530013   0.16560705
##               strategies_8 strategies_9 strategies_10 strategies_11
## strategies_2   0.076134499   0.16856409    0.11753804   -0.16837611
## strategies_3  -0.112668689  -0.09982774    0.15963635    0.27412948
## strategies_4  -0.006399877  -0.03122543   -0.06220908   -0.15311752
## strategies_5  -0.283727886  -0.08921551    0.23432085   -0.24790456
## strategies_6  -0.015756178   0.37182612   -0.15315563    0.02307965
## strategies_8   1.000000000   0.08407508   -0.06429807    0.06649548
## strategies_9   0.084075081   1.00000000   -0.24517188   -0.09733076
## strategies_10 -0.064298066  -0.24517188    1.00000000    0.05817237
## strategies_11  0.066495476  -0.09733076    0.05817237    1.00000000
## strategies_12  0.147194012   0.33709993    0.10219843   -0.02874798
## strategies_13 -0.028464342   0.03306653    0.12297044   -0.04864367
## strategies_14 -0.016487400   0.05536994    0.06701923    0.07172041
##               strategies_12 strategies_13 strategies_14
## strategies_2     0.06776662   -0.09828519   -0.19149091
## strategies_3    -0.03715172   -0.15366614    0.01618328
## strategies_4     0.02075143   -0.08323067   -0.01972215
## strategies_5    -0.00922286    0.16472736    0.21530013
## strategies_6     0.17759507   -0.01463641    0.16560705
## strategies_8     0.14719401   -0.02846434   -0.01648740
## strategies_9     0.33709993    0.03306653    0.05536994
## strategies_10    0.10219843    0.12297044    0.06701923
## strategies_11   -0.02874798   -0.04864367    0.07172041
## strategies_12    1.00000000    0.04785661    0.11663992
## strategies_13    0.04785661    1.00000000    0.14984987
## strategies_14    0.11663992    0.14984987    1.00000000
```

```
paran::paran(strat.comp.fa, cfa = TRUE)
```

```
## 
## Using eigendecomposition of correlation matrix.
## Computing: 10%  20%  30%  40%  50%  60%  70%  80%  90%  100%
## 
## 
## Results of Horn's Parallel Analysis for factor retention
## 360 iterations, using the mean estimate
## 
## -------------------------------------------------- 
## Factor      Adjusted    Unadjusted    Estimated 
##             Eigenvalue  Eigenvalue    Bias 
## -------------------------------------------------- 
## 1           0.158909    1.286568      1.127659
## 2           0.338308    1.190833      0.852524
## 3           0.121592    0.771493      0.649901
## 4           0.187462    0.674199      0.486736
## 5           0.080096    0.404176      0.324079
## 6           0.011533    0.205512      0.193978
## -------------------------------------------------- 
## 
## Adjusted eigenvalues > 0 indicate dimensions to retain.
## (6 factors    retained)
```

```
strat.fa <- psych::fa(strat.comp.fa, nfactors = 6, rotate = "none")

psych::print.psych(strat.fa, cut = .30)
```

```
## Factor Analysis using method =  minres
## Call: psych::fa(r = strat.comp.fa, nfactors = 6, rotate = "none")
## Standardized loadings (pattern matrix) based upon correlation matrix
##                 MR1   MR2   MR3   MR4   MR5   MR6   h2      u2 com
## strategies_2         0.40       -0.48             0.48 0.52278 2.8
## strategies_3   0.75        0.51                   0.94 0.05889 2.2
## strategies_4         0.33       -0.37        0.36 0.42 0.58494 3.6
## strategies_5   0.86       -0.46                   1.00 0.00013 1.7
## strategies_6         0.69                         0.58 0.41854 1.5
## strategies_8                                      0.10 0.90022 2.3
## strategies_9         0.68                         0.76 0.23902 2.5
## strategies_10  0.43                    0.66       0.78 0.21908 2.6
## strategies_11              0.48  0.49             0.54 0.45752 2.6
## strategies_12                          0.34       0.23 0.76992 2.5
## strategies_13                                     0.13 0.86930 2.1
## strategies_14                                0.31 0.28 0.71790 4.0
## 
##                        MR1  MR2  MR3  MR4  MR5  MR6
## SS loadings           1.69 1.46 1.02 0.86 0.76 0.45
## Proportion Var        0.14 0.12 0.09 0.07 0.06 0.04
## Cumulative Var        0.14 0.26 0.35 0.42 0.48 0.52
## Proportion Explained  0.27 0.23 0.16 0.14 0.12 0.07
## Cumulative Proportion 0.27 0.50 0.67 0.81 0.93 1.00
## 
## Mean item complexity =  2.5
## Test of the hypothesis that 6 factors are sufficient.
## 
## The degrees of freedom for the null model are  66  and the objective function was  2.07 with Chi Square of  95.75
## The degrees of freedom for the model are 9  and the objective function was  0.04 
## 
## The root mean square of the residuals (RMSR) is  0.02 
## The df corrected root mean square of the residuals is  0.04 
## 
## The harmonic number of observations is  52 with the empirical chi square  1.57  with prob <  1 
## The total number of observations was  52  with Likelihood Chi Square =  1.7  with prob <  1 
## 
## Tucker Lewis Index of factoring reliability =  3.495
## RMSEA index =  0  and the 90 % confidence intervals are  0 0
## BIC =  -33.86
## Fit based upon off diagonal values = 0.99
## Measures of factor score adequacy             
##                                                    MR1  MR2  MR3  MR4  MR5  MR6
## Correlation of (regression) scores with factors   0.98 0.90 0.97 0.81 0.86 0.72
## Multiple R square of scores with factors          0.97 0.81 0.94 0.66 0.74 0.52
## Minimum correlation of possible factor scores     0.94 0.62 0.88 0.31 0.47 0.04
```

## Emotions

```
cor(emot.comp.fa)
```

```
##              emotions_1  emotions_2  emotions_3 emotions_4  emotions_5
## emotions_1   1.00000000  0.56087225 -0.17263340  0.5747960  0.56556114
## emotions_2   0.56087225  1.00000000 -0.12019738  0.4195112  0.41277122
## emotions_3  -0.17263340 -0.12019738  1.00000000 -0.3710652 -0.21069849
## emotions_4   0.57479602  0.41951124 -0.37106516  1.0000000  0.51869339
## emotions_5   0.56556114  0.41277122 -0.21069849  0.5186934  1.00000000
## emotions_6   0.05036629 -0.17223058  0.40454845 -0.1602365 -0.12349019
## emotions_7  -0.19795660 -0.09439188  0.50059432 -0.2580240 -0.24819460
## emotions_8   0.55767655  0.31790147 -0.17758107  0.4685711  0.59371121
## emotions_9   0.41408316  0.37089436  0.14157548  0.4681206  0.41595915
## emotions_10  0.40795467  0.13672365 -0.11631732  0.1548140  0.02500601
## emotions_11  0.49971069  0.34805650 -0.07295421  0.2165731  0.40347259
## emotions_12  0.44242929  0.39435536  0.14319990  0.2719492  0.25243908
## emotions_13  0.38595235  0.34401515  0.03212233  0.2657903  0.37751101
## emotions_14  0.22303218  0.12340891  0.12946600  0.1419162  0.40876722
##              emotions_6   emotions_7  emotions_8  emotions_9 emotions_10
## emotions_1   0.05036629 -0.197956597  0.55767655  0.41408316  0.40795467
## emotions_2  -0.17223058 -0.094391881  0.31790147  0.37089436  0.13672365
## emotions_3   0.40454845  0.500594320 -0.17758107  0.14157548 -0.11631732
## emotions_4  -0.16023651 -0.258023957  0.46857115  0.46812057  0.15481400
## emotions_5  -0.12349019 -0.248194604  0.59371121  0.41595915  0.02500601
## emotions_6   1.00000000  0.210421276 -0.05518040 -0.01098228  0.25315637
## emotions_7   0.21042128  1.000000000 -0.17071303  0.02616006 -0.14927085
## emotions_8  -0.05518040 -0.170713033  1.00000000  0.37760134  0.23328495
## emotions_9  -0.01098228  0.026160062  0.37760134  1.00000000  0.14232613
## emotions_10  0.25315637 -0.149270848  0.23328495  0.14232613  1.00000000
## emotions_11  0.08740318  0.001123362  0.26273653  0.33988041  0.44310197
## emotions_12  0.07553639 -0.054621452  0.05974075  0.44757508  0.38457660
## emotions_13 -0.08707425  0.025225899  0.15017270  0.60058323  0.13724373
## emotions_14  0.04841591  0.022762075  0.22720336  0.23561914  0.07017548
##              emotions_11 emotions_12 emotions_13 emotions_14
## emotions_1   0.499710691  0.44242929  0.38595235  0.22303218
## emotions_2   0.348056498  0.39435536  0.34401515  0.12340891
## emotions_3  -0.072954207  0.14319990  0.03212233  0.12946600
## emotions_4   0.216573128  0.27194916  0.26579031  0.14191621
## emotions_5   0.403472588  0.25243908  0.37751101  0.40876722
## emotions_6   0.087403183  0.07553639 -0.08707425  0.04841591
## emotions_7   0.001123362 -0.05462145  0.02522590  0.02276207
## emotions_8   0.262736530  0.05974075  0.15017270  0.22720336
## emotions_9   0.339880409  0.44757508  0.60058323  0.23561914
## emotions_10  0.443101971  0.38457660  0.13724373  0.07017548
## emotions_11  1.000000000  0.47596340  0.64010889  0.38367186
## emotions_12  0.475963397  1.00000000  0.36011849  0.20240884
## emotions_13  0.640108888  0.36011849  1.00000000  0.27783961
## emotions_14  0.383671859  0.20240884  0.27783961  1.00000000
```

```
paran::paran(emot.comp.fa, cfa = TRUE)
```

```
## 
## Using eigendecomposition of correlation matrix.
## Computing: 10%  20%  30%  40%  50%  60%  70%  80%  90%  100%
## 
## 
## Results of Horn's Parallel Analysis for factor retention
## 420 iterations, using the mean estimate
## 
## -------------------------------------------------- 
## Factor      Adjusted    Unadjusted    Estimated 
##             Eigenvalue  Eigenvalue    Bias 
## -------------------------------------------------- 
## 1           2.913695    4.211531      1.297835
## 2           0.579820    1.593152      1.013331
## 3           0.050279    0.859447      0.809167
## 4           0.016533    0.646807      0.630274
## 5           0.015175    0.486929      0.471754
## -------------------------------------------------- 
## 
## Adjusted eigenvalues > 0 indicate dimensions to retain.
## (5 factors    retained)
```

```
emot.fa <- psych::fa(emot.comp.fa, nfactors = 5, rotate = "none")

psych::print.psych(emot.fa, cut = .30)
```

```
## Factor Analysis using method =  minres
## Call: psych::fa(r = emot.comp.fa, nfactors = 5, rotate = "none")
## Standardized loadings (pattern matrix) based upon correlation matrix
##               MR1   MR2   MR3   MR4   MR5   h2     u2 com
## emotions_1   0.81                         0.73 0.2739 1.2
## emotions_2   0.59                         0.40 0.6006 1.3
## emotions_3         0.88  0.31             1.00 0.0025 1.6
## emotions_4   0.65 -0.32                   0.59 0.4088 1.9
## emotions_5   0.73        0.32             0.77 0.2278 1.9
## emotions_6         0.44        0.32       0.36 0.6399 2.6
## emotions_7         0.47                   0.28 0.7158 1.6
## emotions_8   0.59              0.37       0.59 0.4145 2.4
## emotions_9   0.64                         0.60 0.3999 2.0
## emotions_10  0.40       -0.67             0.67 0.3338 2.0
## emotions_11  0.72  0.33 -0.31 -0.36  0.30 0.95 0.0547 2.9
## emotions_12  0.54  0.31                   0.49 0.5108 2.4
## emotions_13  0.62             -0.42       0.66 0.3394 2.4
## emotions_14  0.37                    0.31 0.29 0.7107 2.8
## 
##                        MR1  MR2  MR3  MR4  MR5
## SS loadings           4.28 1.78 1.00 0.75 0.56
## Proportion Var        0.31 0.13 0.07 0.05 0.04
## Cumulative Var        0.31 0.43 0.50 0.56 0.60
## Proportion Explained  0.51 0.21 0.12 0.09 0.07
## Cumulative Proportion 0.51 0.72 0.84 0.93 1.00
## 
## Mean item complexity =  2.1
## Test of the hypothesis that 5 factors are sufficient.
## 
## The degrees of freedom for the null model are  91  and the objective function was  6.23 with Chi Square of  283.54
## The degrees of freedom for the model are 31  and the objective function was  0.63 
## 
## The root mean square of the residuals (RMSR) is  0.03 
## The df corrected root mean square of the residuals is  0.06 
## 
## The harmonic number of observations is  52 with the empirical chi square  10.2  with prob <  1 
## The total number of observations was  52  with Likelihood Chi Square =  26.69  with prob <  0.69 
## 
## Tucker Lewis Index of factoring reliability =  1.074
## RMSEA index =  0  and the 90 % confidence intervals are  0 0.084
## BIC =  -95.8
## Fit based upon off diagonal values = 0.99
## Measures of factor score adequacy             
##                                                    MR1  MR2  MR3  MR4  MR5
## Correlation of (regression) scores with factors   0.97 0.99 0.89 0.88 0.81
## Multiple R square of scores with factors          0.94 0.99 0.80 0.77 0.65
## Minimum correlation of possible factor scores     0.88 0.98 0.60 0.53 0.30
```

# Factor scores

```
strat.fs <- psych::factor.scores(strat.comp.fa, strat.fa)$scores
colnames(strat.fs) <- c("strat.1", "strat.2", "strat.3", "strat.4", "strat.5", "strat.6")

emot.fs <- psych::factor.scores(emot.comp.fa, emot.fa)$scores
colnames(emot.fs) <- c("emot.1", "emot.2", "emot.3", "emot.4", "emot.5")

data <- cbind(data, strat.fs, emot.fs)
```

```
cor(select(data, starts_with("strat."), starts_with("emot."), goals_1, goals_2, goals_3, goals_4)) %>% 
  round(3)
```

```
##         strat.1 strat.2 strat.3 strat.4 strat.5 strat.6 emot.1 emot.2 emot.3
## strat.1   1.000   0.000   0.000   0.000   0.000   0.000 -0.172  0.383  0.123
## strat.2   0.000   1.000   0.000   0.000   0.000   0.000  0.381 -0.125 -0.082
## strat.3   0.000   0.000   1.000   0.000   0.000   0.000 -0.129  0.044 -0.114
## strat.4   0.000   0.000   0.000   1.000   0.000   0.000 -0.186 -0.089  0.111
## strat.5   0.000   0.000   0.000   0.000   1.000   0.000  0.190 -0.097  0.033
## strat.6   0.000   0.000   0.000   0.000   0.000   1.000 -0.289  0.037  0.087
## emot.1   -0.172   0.381  -0.129  -0.186   0.190  -0.289  1.000  0.000  0.000
## emot.2    0.383  -0.125   0.044  -0.089  -0.097   0.037  0.000  1.000  0.000
## emot.3    0.123  -0.082  -0.114   0.111   0.033   0.087  0.000  0.000  1.000
## emot.4   -0.189  -0.131   0.074   0.115   0.191  -0.007  0.000  0.000  0.000
## emot.5    0.120  -0.224  -0.174  -0.149  -0.054  -0.013  0.000  0.000  0.000
## goals_1  -0.054   0.248  -0.246   0.196   0.192  -0.061  0.251  0.054  0.121
## goals_2   0.026   0.072   0.083   0.251   0.159   0.085 -0.115 -0.027 -0.019
## goals_3   0.146   0.097  -0.198   0.066   0.134   0.255 -0.159 -0.150  0.086
## goals_4   0.294   0.111   0.032   0.004   0.044   0.266 -0.047  0.295  0.147
##         emot.4 emot.5 goals_1 goals_2 goals_3 goals_4
## strat.1 -0.189  0.120  -0.054   0.026   0.146   0.294
## strat.2 -0.131 -0.224   0.248   0.072   0.097   0.111
## strat.3  0.074 -0.174  -0.246   0.083  -0.198   0.032
## strat.4  0.115 -0.149   0.196   0.251   0.066   0.004
## strat.5  0.191 -0.054   0.192   0.159   0.134   0.044
## strat.6 -0.007 -0.013  -0.061   0.085   0.255   0.266
## emot.1   0.000  0.000   0.251  -0.115  -0.159  -0.047
## emot.2   0.000  0.000   0.054  -0.027  -0.150   0.295
## emot.3   0.000  0.000   0.121  -0.019   0.086   0.147
## emot.4   1.000  0.000  -0.128   0.052  -0.099  -0.008
## emot.5   0.000  1.000  -0.189   0.152   0.156  -0.035
## goals_1 -0.128 -0.189   1.000   0.023   0.067   0.145
## goals_2  0.052  0.152   0.023   1.000   0.478   0.377
## goals_3 -0.099  0.156   0.067   0.478   1.000   0.435
## goals_4 -0.008 -0.035   0.145   0.377   0.435   1.000
```

```
cor.test(data$emot.1, data$strat.2)
```

```
## 
##  Pearson's product-moment correlation
## 
## data:  data$emot.1 and data$strat.2
## t = 2.9161, df = 50, p-value = 0.005294
## alternative hypothesis: true correlation is not equal to 0
## 95 percent confidence interval:
##  0.1209354 0.5925066
## sample estimates:
##       cor 
## 0.3812531
```

```
cor.test(data$emot.2, data$strat.1)
```

```
## 
##  Pearson's product-moment correlation
## 
## data:  data$emot.2 and data$strat.1
## t = 2.9326, df = 50, p-value = 0.00506
## alternative hypothesis: true correlation is not equal to 0
## 95 percent confidence interval:
##  0.1230574 0.5939027
## sample estimates:
##       cor 
## 0.3830925
```

```
cor.test(data$emot.1, data$goals_1)
```

```
## 
##  Pearson's product-moment correlation
## 
## data:  data$emot.1 and data$goals_1
## t = 1.8367, df = 50, p-value = 0.0722
## alternative hypothesis: true correlation is not equal to 0
## 95 percent confidence interval:
##  -0.02307766  0.49064383
## sample estimates:
##      cor 
## 0.251406
```

```
cor.test(data$strat.2, data$goals_1)
```

```
## 
##  Pearson's product-moment correlation
## 
## data:  data$strat.2 and data$goals_1
## t = 1.8129, df = 50, p-value = 0.07586
## alternative hypothesis: true correlation is not equal to 0
## 95 percent confidence interval:
##  -0.02633716  0.48816353
## sample estimates:
##       cor 
## 0.2483481
```
