# Supplementary material for: Swedish and Norwegian Police Interviewers' Goals, Tactics, and Emotions When Interviewing Suspects of Child Sexual Abuse
Source: Front Psychol. 2021 Jul 9;12:606774. doi: 10.3389/fpsyg.2021.606774 (PMC8298905; doi:10.3389/fpsyg.2021.606774)
Supplement: Supplementary file 1 [file Data_Sheet_1.ZIP › EFA_sweden.html]

Factor analysis of reported strategies and reported emotions – Swedish sample


# Factor analysis of reported strategies and reported emotions – Swedish sample

# Imputations for factor analysis

```
set.seed(123)
```

```
strategies <- zap_labels(swe.trunc) %>% 
  select(starts_with("strategies"))

emotions <- zap_labels(swe.trunc) %>% 
  select(starts_with("emotions"))
```

```
strat.impute <- mice(strategies, m = 5, method = "pmm")
```

```
## 
##  iter imp variable
##   1   1  strategies_3  strategies_4  strategies_6  strategies_13
##   1   2  strategies_3  strategies_4  strategies_6  strategies_13
##   1   3  strategies_3  strategies_4  strategies_6  strategies_13
##   1   4  strategies_3  strategies_4  strategies_6  strategies_13
##   1   5  strategies_3  strategies_4  strategies_6  strategies_13
##   2   1  strategies_3  strategies_4  strategies_6  strategies_13
##   2   2  strategies_3  strategies_4  strategies_6  strategies_13
##   2   3  strategies_3  strategies_4  strategies_6  strategies_13
##   2   4  strategies_3  strategies_4  strategies_6  strategies_13
##   2   5  strategies_3  strategies_4  strategies_6  strategies_13
##   3   1  strategies_3  strategies_4  strategies_6  strategies_13
##   3   2  strategies_3  strategies_4  strategies_6  strategies_13
##   3   3  strategies_3  strategies_4  strategies_6  strategies_13
##   3   4  strategies_3  strategies_4  strategies_6  strategies_13
##   3   5  strategies_3  strategies_4  strategies_6  strategies_13
##   4   1  strategies_3  strategies_4  strategies_6  strategies_13
##   4   2  strategies_3  strategies_4  strategies_6  strategies_13
##   4   3  strategies_3  strategies_4  strategies_6  strategies_13
##   4   4  strategies_3  strategies_4  strategies_6  strategies_13
##   4   5  strategies_3  strategies_4  strategies_6  strategies_13
##   5   1  strategies_3  strategies_4  strategies_6  strategies_13
##   5   2  strategies_3  strategies_4  strategies_6  strategies_13
##   5   3  strategies_3  strategies_4  strategies_6  strategies_13
##   5   4  strategies_3  strategies_4  strategies_6  strategies_13
##   5   5  strategies_3  strategies_4  strategies_6  strategies_13
```

```
strat.comp <- complete(strat.impute)

strat.comp.fa <- strat.comp
```

```
emot.impute <- mice(emotions, m = 5, method = "pmm")
```

```
## 
##  iter imp variable
##   1   1  emotions_3  emotions_7  emotions_8  emotions_11
##   1   2  emotions_3  emotions_7  emotions_8  emotions_11
##   1   3  emotions_3  emotions_7  emotions_8  emotions_11
##   1   4  emotions_3  emotions_7  emotions_8  emotions_11
##   1   5  emotions_3  emotions_7  emotions_8  emotions_11
##   2   1  emotions_3  emotions_7  emotions_8  emotions_11
##   2   2  emotions_3  emotions_7  emotions_8  emotions_11
##   2   3  emotions_3  emotions_7  emotions_8  emotions_11
##   2   4  emotions_3  emotions_7  emotions_8  emotions_11
##   2   5  emotions_3  emotions_7  emotions_8  emotions_11
##   3   1  emotions_3  emotions_7  emotions_8  emotions_11
##   3   2  emotions_3  emotions_7  emotions_8  emotions_11
##   3   3  emotions_3  emotions_7  emotions_8  emotions_11
##   3   4  emotions_3  emotions_7  emotions_8  emotions_11
##   3   5  emotions_3  emotions_7  emotions_8  emotions_11
##   4   1  emotions_3  emotions_7  emotions_8  emotions_11
##   4   2  emotions_3  emotions_7  emotions_8  emotions_11
##   4   3  emotions_3  emotions_7  emotions_8  emotions_11
##   4   4  emotions_3  emotions_7  emotions_8  emotions_11
##   4   5  emotions_3  emotions_7  emotions_8  emotions_11
##   5   1  emotions_3  emotions_7  emotions_8  emotions_11
##   5   2  emotions_3  emotions_7  emotions_8  emotions_11
##   5   3  emotions_3  emotions_7  emotions_8  emotions_11
##   5   4  emotions_3  emotions_7  emotions_8  emotions_11
##   5   5  emotions_3  emotions_7  emotions_8  emotions_11
```

```
emot.comp <- complete(emot.impute)

emot.comp.fa <- emot.comp
```

# Factor analysis

## Strategies

```
cor(strat.comp.fa)
```

```
##               strategies_1 strategies_2 strategies_3 strategies_4 strategies_5
## strategies_1   1.000000000 -0.169515876   0.32500000  -0.10504515  -0.05342716
## strategies_2  -0.169515876  1.000000000  -0.10170953   0.27934449   0.03889076
## strategies_3   0.325000000 -0.101709526   1.00000000  -0.12309978   0.09428323
## strategies_4  -0.105045146  0.279344488  -0.12309978   1.00000000  -0.02063332
## strategies_5  -0.053427165  0.038890761   0.09428323  -0.02063332   1.00000000
## strategies_6  -0.118995294  0.120753225  -0.02383981   0.00428078  -0.01995389
## strategies_7   0.080582296 -0.105181835   0.04230571   0.07406682  -0.07800161
## strategies_8   0.055331270  0.009379529   0.04979814  -0.09125282   0.11181365
## strategies_9  -0.109953537  0.086864168  -0.04745636   0.05829524   0.16798739
## strategies_10  0.040708383 -0.197533030   0.01717385  -0.32940242   0.07228455
## strategies_11  0.230158582 -0.105899306   0.25481843  -0.16405850   0.01498340
## strategies_12 -0.228914643  0.159413764  -0.10208356   0.11616997   0.08292855
## strategies_13  0.004826639  0.153001903  -0.00361998   0.15939285   0.08593260
## strategies_14 -0.184998916  0.199921615  -0.33675584   0.11310752   0.14535246
##               strategies_6 strategies_7 strategies_8 strategies_9 strategies_10
## strategies_1   -0.11899529   0.08058230  0.055331270  -0.10995354   0.040708383
## strategies_2    0.12075323  -0.10518183  0.009379529   0.08686417  -0.197533030
## strategies_3   -0.02383981   0.04230571  0.049798143  -0.04745636   0.017173849
## strategies_4    0.00428078   0.07406682 -0.091252816   0.05829524  -0.329402422
## strategies_5   -0.01995389  -0.07800161  0.111813648   0.16798739   0.072284554
## strategies_6    1.00000000   0.07080034  0.291597143   0.22869019   0.113554471
## strategies_7    0.07080034   1.00000000  0.121098856  -0.27968483   0.401845936
## strategies_8    0.29159714   0.12109886  1.000000000   0.19957385   0.006644717
## strategies_9    0.22869019  -0.27968483  0.199573854   1.00000000  -0.216728562
## strategies_10   0.11355447   0.40184594  0.006644717  -0.21672856   1.000000000
## strategies_11   0.25913886   0.07418683  0.106609863   0.04877177   0.219176655
## strategies_12   0.19055734  -0.05583796  0.126592913   0.30618549  -0.100743189
## strategies_13   0.03520828   0.07623257  0.001548972   0.08045704   0.015964380
## strategies_14   0.14795368   0.02608837  0.137677225   0.18878056  -0.039420113
##               strategies_11 strategies_12 strategies_13 strategies_14
## strategies_1     0.23015858   -0.22891464   0.004826639   -0.18499892
## strategies_2    -0.10589931    0.15941376   0.153001903    0.19992161
## strategies_3     0.25481843   -0.10208356  -0.003619980   -0.33675584
## strategies_4    -0.16405850    0.11616997   0.159392852    0.11310752
## strategies_5     0.01498340    0.08292855   0.085932604    0.14535246
## strategies_6     0.25913886    0.19055734   0.035208280    0.14795368
## strategies_7     0.07418683   -0.05583796   0.076232570    0.02608837
## strategies_8     0.10660986    0.12659291   0.001548972    0.13767722
## strategies_9     0.04877177    0.30618549   0.080457042    0.18878056
## strategies_10    0.21917665   -0.10074319   0.015964380   -0.03942011
## strategies_11    1.00000000   -0.06000991   0.042055215   -0.09504261
## strategies_12   -0.06000991    1.00000000  -0.114520192    0.24822786
## strategies_13    0.04205521   -0.11452019   1.000000000    0.20146576
## strategies_14   -0.09504261    0.24822786   0.201465762    1.00000000
```

```
paran::paran(strat.comp.fa, cfa = TRUE)
```

```
## 
## Using eigendecomposition of correlation matrix.
## Computing: 10%  20%  30%  40%  50%  60%  70%  80%  90%  100%
## 
## 
## Results of Horn's Parallel Analysis for factor retention
## 420 iterations, using the mean estimate
## 
## -------------------------------------------------- 
## Factor      Adjusted    Unadjusted    Estimated 
##             Eigenvalue  Eigenvalue    Bias 
## -------------------------------------------------- 
## No components passed. 
## -------------------------------------------------- 
## 1           0.890393    1.621887      0.731494
## 2           0.470331    1.039173      0.568842
## 3           0.308957    0.759273      0.450316
## 4           0.144128    0.496446      0.352317
## 5           0.042616    0.300743      0.258126
## -------------------------------------------------- 
## 
## Adjusted eigenvalues > 0 indicate dimensions to retain.
## (5 factors    retained)
```

```
strat.fa <- psych::fa(strat.comp.fa, nfactors = 6, rotate = "none")

psych::print.psych(strat.fa, cut = .30)
```

```
## Factor Analysis using method =  minres
## Call: psych::fa(r = strat.comp.fa, nfactors = 6, rotate = "none")
## Standardized loadings (pattern matrix) based upon correlation matrix
##                 MR1   MR2   MR3   MR4   MR5   MR6   h2      u2 com
## strategies_1  -0.40                               0.27 7.3e-01 2.3
## strategies_2   0.40                               0.21 7.9e-01 1.7
## strategies_3  -0.38       -0.37  0.36             0.46 5.4e-01 3.6
## strategies_4   0.36        0.33  0.35             0.46 5.4e-01 4.1
## strategies_5                           0.30       0.15 8.5e-01 2.4
## strategies_6   0.31  0.81                         1.00 2.4e-03 2.2
## strategies_7  -0.45  0.53  0.66                   1.00 4.1e-05 3.2
## strategies_8         0.35                         0.20 8.0e-01 2.3
## strategies_9   0.47                               0.39 6.1e-01 2.6
## strategies_10 -0.44  0.39                         0.51 4.9e-01 3.6
## strategies_11        0.33                         0.28 7.2e-01 3.4
## strategies_12  0.44                          0.32 0.35 6.5e-01 2.5
## strategies_13                    0.50  0.35       0.51 4.9e-01 3.1
## strategies_14  0.46                               0.39 6.1e-01 2.8
## 
##                        MR1  MR2  MR3  MR4  MR5  MR6
## SS loadings           1.81 1.44 1.11 0.73 0.59 0.49
## Proportion Var        0.13 0.10 0.08 0.05 0.04 0.04
## Cumulative Var        0.13 0.23 0.31 0.36 0.41 0.44
## Proportion Explained  0.29 0.23 0.18 0.12 0.10 0.08
## Cumulative Proportion 0.29 0.53 0.71 0.82 0.92 1.00
## 
## Mean item complexity =  2.8
## Test of the hypothesis that 6 factors are sufficient.
## 
## The degrees of freedom for the null model are  91  and the objective function was  1.92 with Chi Square of  229.86
## The degrees of freedom for the model are 22  and the objective function was  0.15 
## 
## The root mean square of the residuals (RMSR) is  0.03 
## The df corrected root mean square of the residuals is  0.05 
## 
## The harmonic number of observations is  126 with the empirical chi square  14.44  with prob <  0.89 
## The total number of observations was  126  with Likelihood Chi Square =  16.93  with prob <  0.77 
## 
## Tucker Lewis Index of factoring reliability =  1.16
## RMSEA index =  0  and the 90 % confidence intervals are  0 0.053
## BIC =  -89.47
## Fit based upon off diagonal values = 0.97
## Measures of factor score adequacy             
##                                                    MR1  MR2  MR3  MR4  MR5  MR6
## Correlation of (regression) scores with factors   0.90 0.97 0.92 0.76 0.77 0.76
## Multiple R square of scores with factors          0.80 0.95 0.84 0.57 0.59 0.58
## Minimum correlation of possible factor scores     0.61 0.89 0.68 0.15 0.17 0.15
```

## Emotions

```
cor(emot.comp.fa)
```

```
##               emotions_1  emotions_2  emotions_3  emotions_4   emotions_5
## emotions_1   1.000000000  0.53051106  0.07614512  0.32539005  0.611231097
## emotions_2   0.530511056  1.00000000 -0.01562500  0.29805309  0.396014490
## emotions_3   0.076145119 -0.01562500  1.00000000 -0.11186770 -0.116974577
## emotions_4   0.325390049  0.29805309 -0.11186770  1.00000000  0.447766626
## emotions_5   0.611231097  0.39601449 -0.11697458  0.44776663  1.000000000
## emotions_6  -0.053845500 -0.07125813  0.08580666  0.06948569 -0.002305038
## emotions_7  -0.132231236 -0.14514648  0.51364204 -0.18448581 -0.257194761
## emotions_8   0.385046381  0.25411162 -0.07095612  0.32807117  0.449830927
## emotions_9   0.327897162  0.24986272  0.03194592  0.24676851  0.298901113
## emotions_10  0.003778437  0.01309457 -0.10475656  0.19471362  0.134929312
## emotions_11  0.093073768  0.15126806 -0.21874548  0.20573711  0.339539361
## emotions_12  0.081164046  0.14875517 -0.19924178  0.09627812  0.254513213
## emotions_13  0.301669412  0.23095994  0.10508824  0.25824728  0.380095285
## emotions_14  0.377446320  0.62592929 -0.13618023  0.18514396  0.373689342
##               emotions_6  emotions_7  emotions_8 emotions_9  emotions_10
## emotions_1  -0.053845500 -0.13223124  0.38504638 0.32789716  0.003778437
## emotions_2  -0.071258128 -0.14514648  0.25411162 0.24986272  0.013094570
## emotions_3   0.085806663  0.51364204 -0.07095612 0.03194592 -0.104756560
## emotions_4   0.069485686 -0.18448581  0.32807117 0.24676851  0.194713617
## emotions_5  -0.002305038 -0.25719476  0.44983093 0.29890111  0.134929312
## emotions_6   1.000000000  0.11498145  0.05250232 0.11297480  0.172155945
## emotions_7   0.114981450  1.00000000 -0.16999231 0.05759177  0.063115284
## emotions_8   0.052502322 -0.16999231  1.00000000 0.09617533  0.132959923
## emotions_9   0.112974798  0.05759177  0.09617533 1.00000000  0.219437710
## emotions_10  0.172155945  0.06311528  0.13295992 0.21943771  1.000000000
## emotions_11 -0.025204023 -0.17428358  0.08409931 0.25290900  0.467699532
## emotions_12  0.119452535 -0.21047619 -0.01416301 0.33770915  0.418193209
## emotions_13  0.233969462  0.01639235  0.12085387 0.49631706  0.198708248
## emotions_14 -0.080685212 -0.30547267  0.24509878 0.20444041 -0.026502925
##             emotions_11 emotions_12 emotions_13 emotions_14
## emotions_1   0.09307377  0.08116405  0.30166941  0.37744632
## emotions_2   0.15126806  0.14875517  0.23095994  0.62592929
## emotions_3  -0.21874548 -0.19924178  0.10508824 -0.13618023
## emotions_4   0.20573711  0.09627812  0.25824728  0.18514396
## emotions_5   0.33953936  0.25451321  0.38009528  0.37368934
## emotions_6  -0.02520402  0.11945254  0.23396946 -0.08068521
## emotions_7  -0.17428358 -0.21047619  0.01639235 -0.30547267
## emotions_8   0.08409931 -0.01416301  0.12085387  0.24509878
## emotions_9   0.25290900  0.33770915  0.49631706  0.20444041
## emotions_10  0.46769953  0.41819321  0.19870825 -0.02650293
## emotions_11  1.00000000  0.57152437  0.23245399  0.23872551
## emotions_12  0.57152437  1.00000000  0.28136929  0.12213304
## emotions_13  0.23245399  0.28136929  1.00000000  0.22458349
## emotions_14  0.23872551  0.12213304  0.22458349  1.00000000
```

```
paran::paran(emot.comp.fa, cfa = TRUE)
```

```
## 
## Using eigendecomposition of correlation matrix.
## Computing: 10%  20%  30%  40%  50%  60%  70%  80%  90%  100%
## 
## 
## Results of Horn's Parallel Analysis for factor retention
## 420 iterations, using the mean estimate
## 
## -------------------------------------------------- 
## Factor      Adjusted    Unadjusted    Estimated 
##             Eigenvalue  Eigenvalue    Bias 
## -------------------------------------------------- 
## No components passed. 
## -------------------------------------------------- 
## 1           2.560498    3.287090      0.726591
## 2           0.785051    1.351920      0.566869
## 3           0.665811    1.115217      0.449405
## 4           0.161353    0.509036      0.347682
## 5           0.001691    0.259457      0.257766
## 6           0.002080    0.171916      0.169836
## -------------------------------------------------- 
## 
## Adjusted eigenvalues > 0 indicate dimensions to retain.
## (6 factors    retained)
```

```
emot.fa <- psych::fa(emot.comp.fa, nfactors = 6, rotate = "none")

psych::print.psych(emot.fa, cut = .30)
```

```
## Factor Analysis using method =  minres
## Call: psych::fa(r = emot.comp.fa, nfactors = 6, rotate = "none")
## Standardized loadings (pattern matrix) based upon correlation matrix
##               MR1   MR2   MR3   MR4   MR5   MR6   h2     u2 com
## emotions_1   0.63 -0.35                         0.61 0.3934 2.1
## emotions_2   0.69 -0.45        0.51             1.00 0.0018 3.0
## emotions_3               0.57                   0.38 0.6189 1.3
## emotions_4   0.50                               0.32 0.6755 1.6
## emotions_5   0.77             -0.30             0.74 0.2594 1.6
## emotions_6                                      0.22 0.7751 3.7
## emotions_7  -0.36        0.78                   0.86 0.1432 1.8
## emotions_8   0.44             -0.35             0.43 0.5655 3.3
## emotions_9   0.49        0.30                   0.42 0.5834 2.6
## emotions_10  0.31  0.59                         0.57 0.4259 2.4
## emotions_11  0.52  0.52                         0.68 0.3227 3.0
## emotions_12  0.45  0.55                         0.56 0.4359 2.4
## emotions_13  0.52        0.38       -0.39       0.62 0.3829 3.2
## emotions_14  0.56                               0.46 0.5359 2.0
## 
##                        MR1  MR2  MR3  MR4  MR5  MR6
## SS loadings           3.46 1.53 1.35 0.71 0.50 0.33
## Proportion Var        0.25 0.11 0.10 0.05 0.04 0.02
## Cumulative Var        0.25 0.36 0.45 0.50 0.54 0.56
## Proportion Explained  0.44 0.19 0.17 0.09 0.06 0.04
## Cumulative Proportion 0.44 0.63 0.80 0.89 0.96 1.00
## 
## Mean item complexity =  2.4
## Test of the hypothesis that 6 factors are sufficient.
## 
## The degrees of freedom for the null model are  91  and the objective function was  4.34 with Chi Square of  518.78
## The degrees of freedom for the model are 22  and the objective function was  0.18 
## 
## The root mean square of the residuals (RMSR) is  0.02 
## The df corrected root mean square of the residuals is  0.04 
## 
## The harmonic number of observations is  126 with the empirical chi square  8.44  with prob <  1 
## The total number of observations was  126  with Likelihood Chi Square =  20.71  with prob <  0.54 
## 
## Tucker Lewis Index of factoring reliability =  1.013
## RMSEA index =  0  and the 90 % confidence intervals are  0 0.07
## BIC =  -85.69
## Fit based upon off diagonal values = 0.99
## Measures of factor score adequacy             
##                                                    MR1  MR2  MR3  MR4  MR5
## Correlation of (regression) scores with factors   0.96 0.91 0.92 0.89 0.76
## Multiple R square of scores with factors          0.93 0.83 0.84 0.79 0.59
## Minimum correlation of possible factor scores     0.86 0.66 0.68 0.58 0.17
##                                                     MR6
## Correlation of (regression) scores with factors    0.69
## Multiple R square of scores with factors           0.48
## Minimum correlation of possible factor scores     -0.04
```

# Factor scores

```
strat.fs <- psych::factor.scores(strat.comp.fa, strat.fa)$scores
colnames(strat.fs) <- c("strat.1", "strat.2", "strat.3", "strat.4", "strat.5", "strat.6")

emot.fs <- psych::factor.scores(emot.comp.fa, emot.fa)$scores
colnames(emot.fs) <- c("emot.1", "emot.2", "emot.3", "emot.4", "emot.5", "emot.6")

swe.data <- cbind(swedish, strat.fs, emot.fs)
```

```
cor(select(swe.data, starts_with("strat."), starts_with("emot."), goals_1, goals_2, goals_3, goals_4), use = "pairwise.complete") %>% 
  round(3)
```

```
##         strat.1 strat.2 strat.3 strat.4 strat.5 strat.6 emot.1 emot.2 emot.3
## strat.1   1.000   0.000   0.000   0.000   0.000   0.000  0.206 -0.250 -0.128
## strat.2   0.000   1.000   0.000   0.000   0.000   0.000 -0.073 -0.202  0.147
## strat.3   0.000   0.000   1.000   0.000   0.000   0.000 -0.020 -0.240 -0.059
## strat.4   0.000   0.000   0.000   1.000   0.000   0.000  0.109 -0.023  0.120
## strat.5   0.000   0.000   0.000   0.000   1.000   0.000  0.171 -0.090  0.011
## strat.6   0.000   0.000   0.000   0.000   0.000   1.000  0.014  0.037  0.154
## emot.1    0.206  -0.073  -0.020   0.109   0.171   0.014  1.000  0.000  0.000
## emot.2   -0.250  -0.202  -0.240  -0.023  -0.090   0.037  0.000  1.000  0.000
## emot.3   -0.128   0.147  -0.059   0.120   0.011   0.154  0.000  0.000  1.000
## emot.4    0.007  -0.057  -0.196   0.026   0.023   0.008  0.000  0.000  0.000
## emot.5    0.129  -0.038  -0.034   0.088   0.026   0.139  0.000  0.000  0.000
## emot.6   -0.058  -0.127   0.028   0.090  -0.039   0.288  0.000  0.000  0.000
## goals_1   0.222   0.175   0.010   0.118   0.132   0.061  0.084 -0.232  0.075
## goals_2  -0.193   0.103  -0.048   0.058   0.138  -0.001  0.099  0.103  0.148
## goals_3   0.082   0.035  -0.030  -0.044   0.101   0.177  0.001  0.126  0.119
## goals_4   0.072   0.138  -0.107  -0.015  -0.023   0.087  0.070  0.017  0.108
##         emot.4 emot.5 emot.6 goals_1 goals_2 goals_3 goals_4
## strat.1  0.007  0.129 -0.058   0.222  -0.193   0.082   0.072
## strat.2 -0.057 -0.038 -0.127   0.175   0.103   0.035   0.138
## strat.3 -0.196 -0.034  0.028   0.010  -0.048  -0.030  -0.107
## strat.4  0.026  0.088  0.090   0.118   0.058  -0.044  -0.015
## strat.5  0.023  0.026 -0.039   0.132   0.138   0.101  -0.023
## strat.6  0.008  0.139  0.288   0.061  -0.001   0.177   0.087
## emot.1   0.000  0.000  0.000   0.084   0.099   0.001   0.070
## emot.2   0.000  0.000  0.000  -0.232   0.103   0.126   0.017
## emot.3   0.000  0.000  0.000   0.075   0.148   0.119   0.108
## emot.4   1.000  0.000  0.000  -0.172  -0.098  -0.093  -0.024
## emot.5   0.000  1.000  0.000   0.003   0.108   0.041   0.094
## emot.6   0.000  0.000  1.000  -0.105  -0.115  -0.084  -0.121
## goals_1 -0.172  0.003 -0.105   1.000   0.145   0.155   0.178
## goals_2 -0.098  0.108 -0.115   0.145   1.000   0.390   0.428
## goals_3 -0.093  0.041 -0.084   0.155   0.390   1.000   0.357
## goals_4 -0.024  0.094 -0.121   0.178   0.428   0.357   1.000
```

```
cor.test(swe.data$emot.1, swe.data$strat.1)
```

```
## 
##  Pearson's product-moment correlation
## 
## data:  swe.data$emot.1 and swe.data$strat.1
## t = 2.3463, df = 124, p-value = 0.02055
## alternative hypothesis: true correlation is not equal to 0
## 95 percent confidence interval:
##  0.03244283 0.36782220
## sample estimates:
##       cor 
## 0.2061798
```

```
cor.test(swe.data$emot.2, swe.data$goals_1)
```

```
## 
##  Pearson's product-moment correlation
## 
## data:  swe.data$emot.2 and swe.data$goals_1
## t = -2.65, df = 124, p-value = 0.009099
## alternative hypothesis: true correlation is not equal to 0
## 95 percent confidence interval:
##  -0.39059887 -0.05898996
## sample estimates:
##        cor 
## -0.2315082
```

```
cor.test(swe.data$emot.2, swe.data$strat.1)
```

```
## 
##  Pearson's product-moment correlation
## 
## data:  swe.data$emot.2 and swe.data$strat.1
## t = -2.8727, df = 124, p-value = 0.004789
## alternative hypothesis: true correlation is not equal to 0
## 95 percent confidence interval:
##  -0.4069250 -0.0783117
## sample estimates:
##        cor 
## -0.2497971
```

```
cor.test(swe.data$emot.2, swe.data$strat.2)
```

```
## 
##  Pearson's product-moment correlation
## 
## data:  swe.data$emot.2 and swe.data$strat.2
## t = -2.2956, df = 124, p-value = 0.02338
## alternative hypothesis: true correlation is not equal to 0
## 95 percent confidence interval:
##  -0.36396243 -0.02799052
## sample estimates:
##        cor 
## -0.2019089
```

```
cor.test(swe.data$emot.2, swe.data$strat.3)
```

```
## 
##  Pearson's product-moment correlation
## 
## data:  swe.data$emot.2 and swe.data$strat.3
## t = -2.7564, df = 124, p-value = 0.006726
## alternative hypothesis: true correlation is not equal to 0
## 95 percent confidence interval:
##  -0.39844341 -0.06824298
## sample estimates:
##        cor 
## -0.2402819
```
